# Evolution of the Cell Wall Gene Families of Grasses

**DOI:** 10.3389/fpls.2019.01205

**Published:** 2019-10-04

**Authors:** Bryan W. Penning, Maureen C. McCann, Nicholas C. Carpita

**Affiliations:** ^1^Corn, Soybean and Wheat Quality Research, USDA-ARS, Wooster, OH, United States; ^2^Department of Biological Sciences, Purdue University, West Lafayette, IN, United States; ^3^Purdue Center for Plant Biology, West Lafayette, IN, United States; ^4^Department of Botany & Plant Pathology, Purdue University, West Lafayette, IN, United States

**Keywords:** maize (*Zea mays*), cell-wall structure, cell-wall synthesis, type II cell walls, gene annotation, gene expression, stem development

## Abstract

Grasses and related commelinid monocot species synthesize cell walls distinct in composition from other angiosperm species. With few exceptions, the genomes of all angiosperms contain the genes that encode the enzymes for synthesis of all cell-wall polysaccharide, phenylpropanoid, and protein constituents known in vascular plants. RNA-seq analysis of transcripts expressed during development of the upper and lower internodes of maize (*Zea mays*) stem captured the expression of cell-wall-related genes associated with primary or secondary wall formation. High levels of transcript abundances were not confined to genes associated with the distinct walls of grasses but also of those associated with xyloglucan and pectin synthesis. Combined with proteomics data to confirm that expressed genes are translated, we propose that the distinctive cell-wall composition of grasses results from sorting downstream from their sites of synthesis in the Golgi apparatus and hydrolysis of the uncharacteristic polysaccharides and not from differential expression of synthases of grass-specific polysaccharides.

## Introduction

The primary walls of all angiosperms are assembled from scaffolds of cellulose microfibrils interlaced with hemicellulosic xyloglucans (XyGs), (glucuronoarabino)xylans (GAXs), and (gluco)mannans (Scheller and Ulvskov, 2010), and embedded in an independent but coextensive matrix of pectic polysaccharides (McCann and Roberts, 1991; Caffall and Mohnen, 2009). Primary cell walls of angiosperm species are classified into two types (Carpita and Gibeaut, 1993). XyGs are the major hemicellulose in the type I walls of dicots and noncommelinid monocots and contain an abundance of HG and RG-I pectic polysaccharides (Caffall and Mohnen, 2009; Scheller and Ulvskov, 2010). In contrast, type II walls of grasses and other commelinid monocots contain mostly hemicellulosic GAX of varying degrees of side branching but have little pectin (Carpita and Gibeaut, 1993). A (1→3),(1→4)-β-D-glucan (mixed-linkage β-glucan) is found in *Poales* at certain stages of primary wall formation. Small amounts of XyG are also found with side chains terminated by galactose and no detectable Fuc (Carpita et al., 2001). Type I walls incorporate predominantly Hyp- and Gly-rich structural proteins at the end of growth to reinforce the wall into final shape, whereas this reinforcement is supplied by a phenylpropanoid network in the type II wall (Carpita, 1996). This distinction results in strong autofluorescence in the primary wall in addition to that seen in vascular tissue (Rudall and Caddick, 1994). A fraction of the phenolic material is saponifiable, yielding ferulic and *p*-coumaric acid and its dimers (Carpita, 1986), but a substantial amount of the aromatic material is nonsaponifiable hydroxycinnamic acids and their ethers (Scalbert et al., 1985). During vascular development and the formation of a thickened rind of the stem, cellulose, glucuronoxylans (GXs), and GAX become embedded in lignin (Carpita, 1996).

The genome sequences of *Arabidopsis* (*Arabidopsis thaliana*; Arabidopsis Genome Initiative, 2000), rice (*Oryza sativa*; International Rice Genome Sequencing Project, 2005), and maize (*Zea mays* cv. B73; Schnable et al., 2009) enabled comparative genomic analyses of cell-wall-related genes of two grass species with a typical eudicot, *Arabidopsis*. About 60% of *Arabidopsis* genes are annotated with predicted functions (Swarbreck et al., 2008). Based on sequence similarities, the Carbohydrate-Active enZYme database comprises families of glycosyl transferases (GTs), glycosyl hydrolases (GHs), and other carbohydrate-metabolizing enzymes (Lombard et al., 2014; http://www.cazy.org/). We annotated over 750 maize homologs of these cell-wall-related genes and assembled them into gene families predicted to function in cell-wall biogenesis (Penning et al., 2009).

We used phylogenetic comparisons of maize and rice gene families with those of *Arabidopsis* to characterize potential divergences that might explain the differences in composition between type I and type II walls. A further distinction in maize compared to other grasses is a recent genome duplication event (Gaut and Doebley, 1997). We find that many of these paralogous genes were retained, but loss of genes resulted in splitting of a single-gene function between paralogs (subfunctionalization), new function in a paralog gene (neofunctionalization), or a combination of both events (subneofunctionalization) to a greater extent in maize than other grasses (Penning et al., 2009). Copy-number and presence–absence variation has resulted in retention of both paralogs, only one of them, or neither of them (Springer et al., 2009; Swanson-Wagner et al., 2010). Since our previous study (Penning et al., 2009), we have developed a more robust annotation of nearly 1,200 maize genes and classified them into cell-wall-related gene families and their respective subgroups. In addition to families of substrate generation, cellulose and polysaccharide synthases, GTs, and cell-wall modifying enzymes, we broadened the inventory of cell-wall-related genes to include many new families involved in polysaccharide side-group construction, proteases, glycosylphosphatidylinositol (GPI)-anchored proteins, glycoprotein synthesis, and signaling. We show here that, with few exceptions, the composition of gene families of any angiosperm species and levels of expression of their members are not clearly correlated with wall composition.

As the gene families had almost equal representation of all genes associated with cell-wall synthesis, we explored whether maize differential expression of these genes was correlated with the type of wall made. We established differential expression profiles of rind tissues from developing maize stem internodes representative of primary wall versus secondary wall formation to classify highly expressed members of these families. From these data, we could establish if *Arabidopsis* homologs closest in sequence were expressed in a similar primary or secondary wall context and, thus, could be considered functional orthologs. The number of potential orthologs with *Arabidopsis* genes, based on common expression during primary or secondary wall formation of the most similar sequences, was limited. However, we found robust expression of maize genes not only encoding synthases of GAX and mixed-linkage glucans but also those encoding synthases of the pectic polysaccharides, RG-I and HG, and of XyGs—polysaccharides that are only minor constituents of the maize cell wall. Although one cannot infer that expression of a gene necessarily results in translation of their corresponding proteins, our recent analyses of the glycome and proteome of maize Golgi demonstrates unequivocally that the relevant biosynthetic enzymes are present and that substantial amounts of the pectins and XyGs accumulate in the cisternae of this organelle (Okekeogbu et al., 2019). Thus, we propose that cell-wall composition is determined by mechanisms in sorting or metabolism of polysaccharides downstream from Golgi-based synthesis.

## Materials and Methods

### Sequence Alignments and Dendrogram Development

Sequence alignments and phylogenetic trees were constructed as described by Penning et al. (2009). Dendrograms were assembled from protein-coding sequences by the neighbor-joining method in ClustalW (Saitou and Nei, 1987; Chenna et al., 2003). The trees were bootstrapped 1,000 times. After the initial multiple alignment, individual clade alignments were checked using Multalin (Corpet, 1988; http://www-archbac.u-psud.fr/genomics/multalin.html). Matches to conserved regions within groups of family clades with suspect alignments were manually checked using InterProScan (Zdobnov and Apweiler, 2001; http://www.ebi.ac.uk/Tools/InterProScan/), and nonmatching members of the families were removed. Dendrograms were drawn using TreeDyn (Chevenet et al., 2006; http://www.treedyn.org/).

### Protein Motif Analyses

Sequence locations and distances were verified using the Gbrowse designed for rice (http://www.jcvi.org), *Arabidopsis* (http://www.arabidopsis.org), and our custom maize annotation database comprising proteins from maize version 2 WGS (www.maizeGDB.org), using the largest representative transcript of each gene. We also used the Phytozome Protein domain FAMilies/Kyoto Encyclopedia of Genes and Genomes descriptions (https://phytozome.jgi.doe.gov/pz/portal.html), which were in general agreement with our maize cell-wall protein database at the Maize Genetics and Genome database, but our manual annotations based on phylogenetic trees constructed with the most similar *Arabidopsis* and rice sequences refined specific gene descriptions. We have augmented the public database at Maize Genetics and Genome for improved annotation of maize cell wall protein families (https://www.maizegdb.org/gbrowse/maize_v2test?l=CellWallGenes;l=Gene_models;q=Chr1:2650000.2699999 ). This resource is also posted at http://cellwall.genomics.purdue.edu .

### Expression Analysis

Seeds of maize (*Z. mays* cv. B73) were obtained from the Maize Genetics Cooperation Center at Champaign, IL, and propagated and grown at the Purdue University Agricultural Center for Research and Education (West Lafayette, IN). Stem elongation began at the fifth-leaf stage and culminated with tassel formation 10 weeks postplanting. At 49-day postplanting, rind tissues of internodes 4–8 from a minimum of three plants were excised aseptically and immediately plunged in liquid N_2_ and pulverized by mortar and pestle under additional liquid N_2_. Approximately 2 mg of ground tissue was incubated with 1 ml of ice-cold TRIzol reagent (Invitrogen, Life Technologies) and extracted according to the manufacturer’s directions. Purified RNA was dissolved in 100 μl of diethyl pyrocarbonate-treated nanopure water, and quality and concentration were determined spectrophotometrically. Internode 7 failed a quality control analysis for expression of a set of housekeeping genes (Sekhorn et al., 2011) and was excluded from further analysis.

Sections of each internode were frozen to −80°C in Neg 50 section medium (Richard-Allan Scientific, Kalamazoo, MI) and cross-sectioned to a thickness of 100 μm using a Microm HM550 Cryostat (Richard-Allan Scientific) at −20°C. Sections were thawed, rinsed with water, and stained using 2% w/v Wiesner’s solution (phloroglucinol) in a 1:1 mixture of methanol and 50% HCl (v/v), freshly diluted to 5% in water. Images were taken using a SPOT Insight FireWire 4 Megasample Color Mosaic Camera (SPOT imaging systems, www.spotimaging.com) attached to a Nikon SMZ 1500 stereomicroscope (Nikon Corporation, Kanagawa, Japan) using a variable objective lens set to 10×. Images were captured using SPOT Advanced software version 4.1 (SPOT imaging systems).

Expression analysis was carried out as previously described (Penning et al., 2014). Briefly, pooled RNA samples from three biological replicates were sequenced using an Illumina HiSeq 2000 to process 100 bp × 100 bp libraries of ∼400-bp inserts. High-quality trimmed sequences were mapped to the Maize B73 sequence V2 from Plant GDB (http://www.plantgdb.org) using Bowtie2 (Langmead et al., 2009), except in instances where the reads mapped exactly to two genes due to the high degree of gene duplication in maize. A custom Perl script was used to split these reads between the two loci (Penning et al., 2014). A separate set of Perl scripts was used to add closest *Arabidopsis* gene by sequence with description and expect value to the file. One read per million (20 reads per 20 M reads) or greater was used as a threshold for the detection of transcript (Li et al., 2010; Chang et al., 2012). RNA-seq data are available at https://www.ncbi.nlm.nih.gov/sra/PRJNA522448 (datasets: SRX5387736, SRX5387731, SRX5387711, SRX5387715).

## Results

### Annotation of Gene Function

General gene functions in encoding the enzymes of nucleotide sugar interconversion and transport, of polysaccharide synthases and glycosyl transferases, and those that encode their hydrolases and lyases have been inferred primarily from sequence similarity with bacterial genes of similar functions (Lombard et al., 2014). Bioinformatic approaches have extended predictions of gene function across eukaryotic species as increasing numbers of genomes became available. Phytozome, the Plant Comparative Genomics portal of the Department of Energy’s Joint Genome Institute (https://phytozome.jgi.doe.gosv), provides the plant science community with a large assembly of genomes from the JGI-sequencing initiative and those publicly available from other resources (Goodstein et al., 2012). Gene sets are best annotated using the Phytozome Protein domain FAMilies/Kyoto Encyclopedia of Genes and Genomes platforms and complemented with assignments by InterPro protein analysis tools that more closely predict function. Nevertheless, these remain hypothetical in the absence of biochemical or cell biological characterizations. Functional annotation has been facilitated by the identification of mutants representative of the different subgroups within the large gene families of glycosyl transferases and hydrolases. Some of these show chemical and spectroscopic signatures resulting from modified polymer fine structure that does not otherwise affect plant growth or development (Carpita and McCann, 2015).

The three angiosperms examined, *Arabidopsis*, rice, and maize, had members represented in every family of cell-wall-related genes. Within large families common to grasses and dicots, subgroup structure indicated putative orthology based on high sequence similarity of genes and similar patterns of expression at elongation and primary wall synthesis versus secondary wall synthesis stages of stem development. However, in other subgroups, grasses displayed a marked divergence from *Arabidopsis* in the number of members and degree of sequence similarity of homologs within a family subgroup, or even the presence or absence of grass-specific subgroups. Examples of all three of these characteristics are observed in the subgroup structure of the cellulose synthase (CesA)/cellulose-synthase-like (Csl) superfamily, where putative orthologs can be identified for the CesA and CslD subgroups, divergence of rice and maize CslAs from those of *Arabidopsis*, and grass-unique subgroups of CslF and CslH from *Arabidopsis*-only subgroup CslB (**Figure S1**).

### Gene Expression During Stem Development

**Figure 1:**
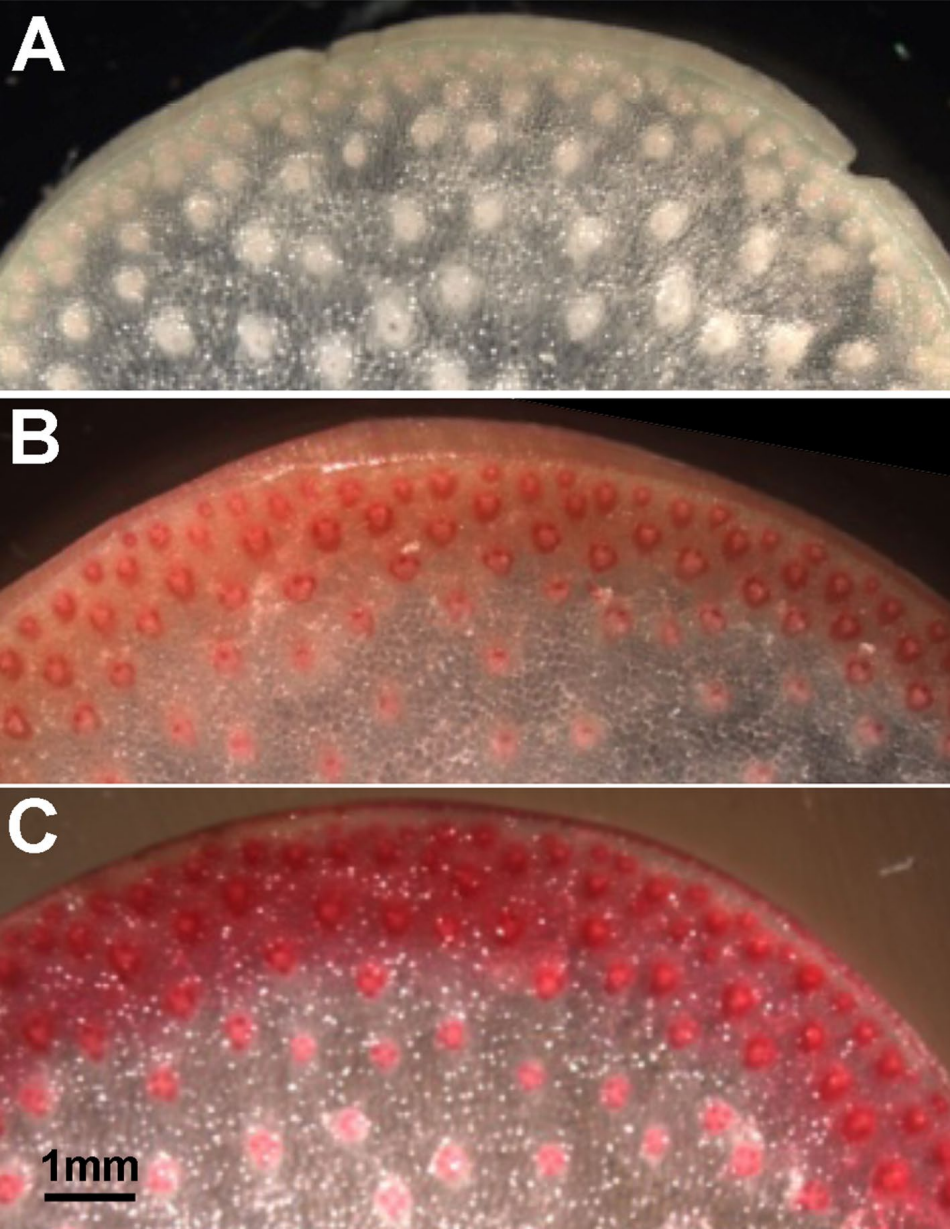
Cell wall thickness and lignin content increase in rind tissues of maize internodes with developmental age. Phloroglucinol staining intensity increases from faint pink to dark red in stem sections from **(A)** an elongation stage internode (internode 8), **(B)** a transitional stage internode (internode 6), and **(C)** a secondary wall stage internode (internode 4). Scale bar, 1 mm.

An estimate of potential functional orthology is obtained by comparative gene expression during the same stages of organ development. For grasses, stem elongation begins in basal internodes and progresses sequentially in upper internodes, culminating with flowering. In field-grown maize, internode development begins about the fifth-leaf stage at ∼35 days postplanting and culminates with tasseling ∼10 weeks. Stem elongation rates peak ∼7 weeks (49 days), when lower internodes 4 and 5 have ceased elongation and the cells of the rind are more actively engaged in secondary wall formation and lignification, while internodes 6 and 8 continue elongation and transition to secondary wall formation. The rind constitutes the outer rings of vascular bundles with fibers to form distinct bands of cells ∼0.5 cm thick that peel from the pith core during late development (**Figure 1**). To evaluate gene expression across the internodes, we used ≥95 reads per 20 M from the four internodes as a minimal criterion of expression. Furthermore, we used ≥500 reads per 20 M as a criterion to evaluate expression ratios from the elongation stages versus secondary wall forming stages of rind development (**Table S1**). From RNA-seq analysis of rind tissues from these four internodes, we found an expression ratio of 2 or higher in transcript abundance in lower internodes compared to upper internodes to be a suitable indicator of expression related to secondary wall formation. Conversely, ratios <1 indicated genes more associated with primary wall formation during internode elongation.

Although an expression ratio of ≥2 was consistent with association with secondary wall formation, a large proportion of the transcript ratios fell between 1 and 2, indicating more complicated expression patterns, which we termed “transitional” from primary wall formation to secondary wall formation (**Table S1**). We also summarized the expression behaviors of families and subgroups of families associated with specific polysaccharides, denoting the genes expressed during stages of primary wall formation, transitional, and secondary wall formation (**Table 1**). In general, genes of nucleotide sugar interconversion and transport are balanced across all three stages. More genes of XyG, glucomannan, mixed-linked β-glucan, and pectin synthesis are expressed during primary wall synthesis stages, but those of GAX are expressed across all stages. By contrast, genes of monolignol and lignin biosynthesis are highly expressed during secondary wall synthesis (**Table 1**).

**Table 1:** Expression profiles of maize genes in family subgroups of cell-wall-related functions.

Cell Wall function	Number of Genes (Number expresssed)^1^	Elongation	Transitional	Secondary

**Sucrose Synthases**	8(8)	4	3	1
**Nucleotide sugar interconversion**	46(39)	12	8	12
**Nucleotide sugar transporters**	65(58)	20	15	15
**Cellulose synthases (CesAs)**	20(19)	6	5	6
**GAX synthesis**				
GT8A (GUX)	7(6)	2	2	0
GT8C (GATL)	10(7)	3	2	1
GT43	16(14)	0	6	6
GT47E	11(10)	4	1	4
GT61	33(22)	7	3	6
**XyG synthesis**				
CslC	8(6)	6	0	0
GT34 (XXT)	18(6)	3	0	0
GT37 (FUT)	17(4)	1	0	1
GT47A	23(5)	3	1	0
**(Gluco)mannan synthesis**				
CslA	10(10)	8	1	0
GT106B (MSR)	6(6)	6	0	0
**Mixed-linkage β-glucan synthesis**				
CslF	9(7)	4	0	1
AGP/N-Glyc/HRGP-like				
AGP/Fasciclin	10(7)	3	0	2
GT31	40(34)	18	6	3
GT77	23(12)	4	1	1
**ER/Golgi resident**	41(37)	27	4	1
**Pectin Synthesis**				
GT8D (GAUT)	23(23)	16	3	2
GT106A(RRT), GT106C(PAGR), and D	16(13)	6	3	1
GT47B	9(8)	6	2	0
**Polygalacturonases**	47(19)	8	3	1
**Acetyl-transferases (TBL/BAHD)**	77(59)	20	13	6
**GPI-anchored proteins (COB/SKU)**	22(15)	9	1	1
**Expansins/XTHs**	87(41)	23	4	4
**Monolignol Synthesis**	100(71)	17	7	30
**Peroxidases/Laccases**	148(67)	26	7	15

^1^Total expression ≥95 reads per 20 M; expression ratios calculated for those with ≥500 reads per 20 M.

Ratio of transcripts from rind tissue of internodes 4–5 (secondary): internodes 6 and 8 (elongation) for genes expressed at ≥500 reads per 20 M were classified as elongation (≤1.04), transitional (1.05–1.94), and secondary (≥1.95) expression. A complete collection of the genes we annotated, including potential Arabidopsis orthologs is given in **Table S1**.

As similar discriminations of gene expression related to primary and secondary wall formation were established in *Arabidopsis* (Brown et al., 2005), putative orthology could be established by function in the same developmental context rather than the homologs most similar in sequence. Using these criteria, we found that only one quarter of maize cell-wall-related genes expressed in stems during secondary wall development were putatively orthologous with those of *Arabidopsis*. A complete index of over 1,200 maize cell-wall-related genes, ratio of secondary/primary wall expression, and putative *Arabidopsis* orthologs is presented (**Table S1**).

### Cellulose Synthase/Cellulose-Synthase-Like Superfamily

**Figure 2:**
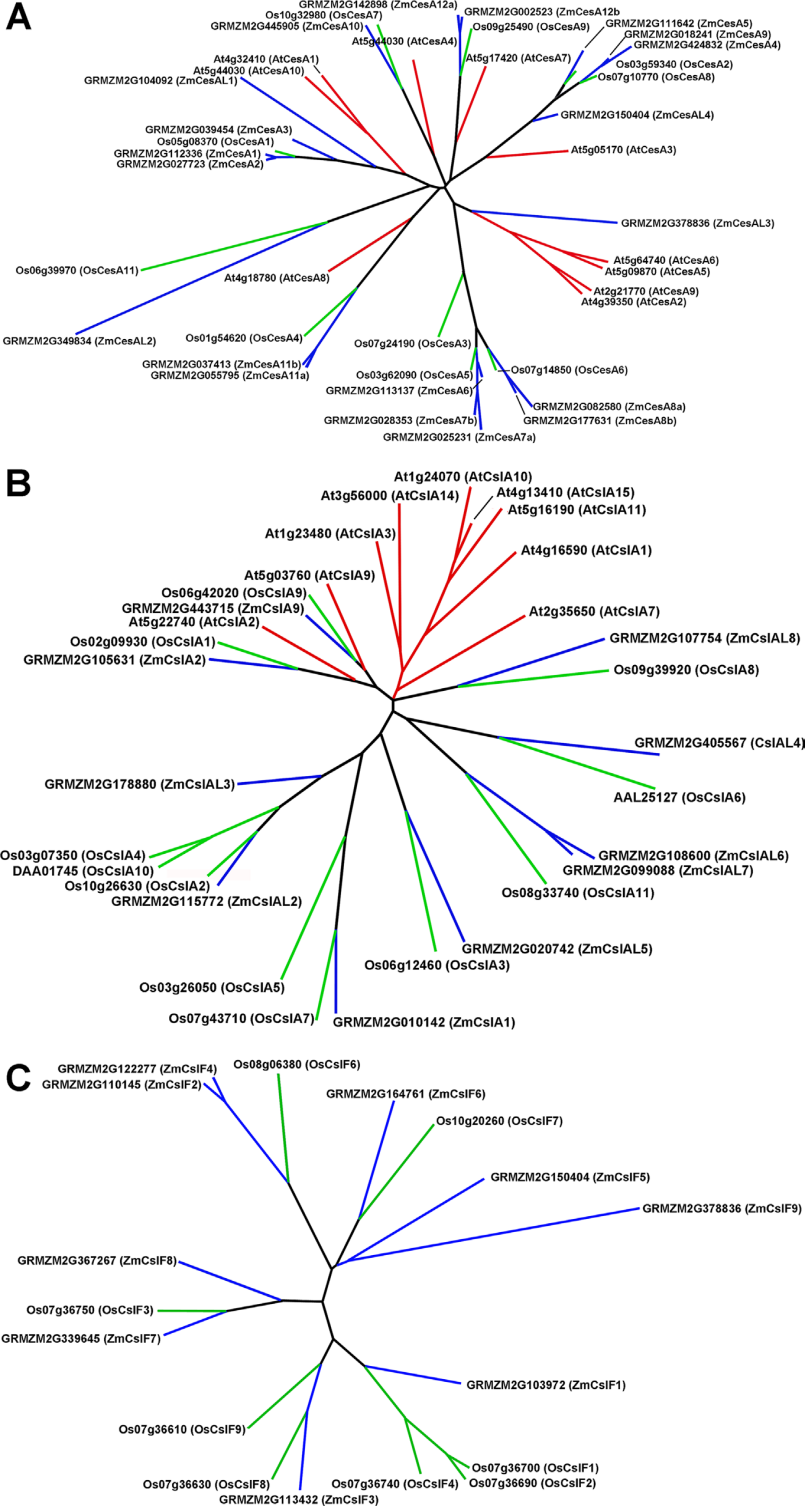
Genes of the cellulose synthase (CesA) and two subgroups of the cellulose-synthase-like (Csl) family for *Arabidopsis*, rice, and maize. **(A)** CesA genes. **(B)** CslA genes. **(C)** CslF genes.

**Figure 3:**
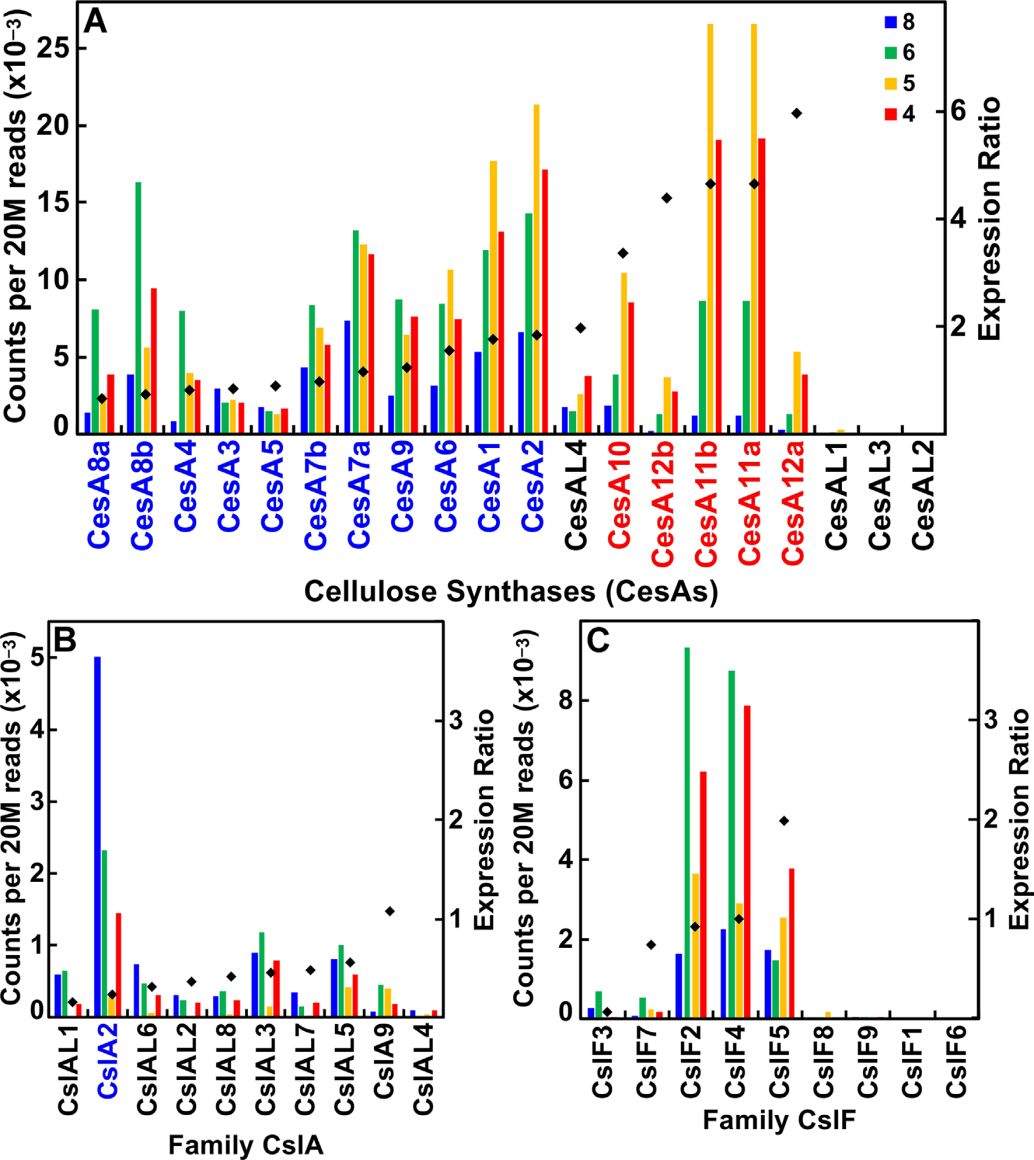
Differential expression of key families of the maize B73 cellulose synthesis and cellulose-synthase-like superfamily. Expression ratio is calculated as the sum of the reads of internodes 4 and 5 divided by the sum of reads of internodes 6 and 8. The expression of maize family member genes is ordered by their ratio of expression in secondary cell wall forming tissue to elongating tissue (diamonds). Putative orthologs of *Arabidopsis* also involved in primary wall (blue) and secondary wall (red) synthesis. **(A)** Cellulose synthase (CesA) genes. **(B)** Cellulose synthase-like subgroup A (CslA) genes. **(C)** Cellulose synthase-like subgroup F (CslF) genes.

The CesA/Csl superfamily comprises up to 10 distinct subfamilies that display divergent membership between grasses and *Arabidopsis*, with the CslB group not represented in the rice or maize genome and the CslF and CslH groups found only in grasses (**Figure S1**). CesA gene families of rice, maize, and *Arabidopsis* show similar subgroup structure (**Figure 2A**), and association within the same subgroup is indicative of a role in primary or secondary wall formation (Tanaka et al., 2003; Appenzeller et al., 2004; Brown et al., 2005). We found that maize CesA1 through CesA9 and their paralogs were associated with elongation and primary wall synthesis stages of development, and CesA10 through CesA12 and their paralogs were associated with secondary wall formation (**Figure 3A**; **Table S1**). From their subgroup membership, those expressed exhibited a ratio of relative secondary to primary wall expression that indicated orthology with *Arabidopsis* for all but one of the 17 CesAs expressed at ≥500 reads per 20 M. Five CesAs whose expression was regarded as “transitional” were homologous with *Arabidopsis* genes of similar sequence that were associated with primary wall formation.

Members of the CslA family in *Arabidopsis* and poplar encode proteins with (gluco)mannan synthase from GDP-Man and GDP-Glc substrates (Dhugga et al., 2004; Liepman et al., 2007). Apart from a few exceptions, maize and rice CslA genes diverged markedly in sequence similarity from *Arabidopsis* genes (**Figure 2B**; **Figure 3B**). All 10 maize CslA genes were expressed at ≥95 reads per 20 M, but expression was either constitutive or higher during primary wall formation (**Figure 3B**; **Table S1**). However, because of the divergence from *Arabidopsis* of the genes in this family, only a single CslA2 gene could be considered potentially orthologous.

Among angiosperm species, the mixed-linkage β-glucans were found only in abundance in the cell walls of *Poales* species (Smith and Harris, 1999), but more recent studies reported immunocytochemical evidence for small amounts of these β-glucans in species outside the Order Poales (Trethewey et al., 2005). The CslF and CslH genes that encode the synthases of the mixed-linkage β-glucans are found only in grass species (Burton et al., 2006; Doblin et al., 2009; Little et al., 2018). Subgroups CslJ and CslM have also been defined in the grasses but are represented outside the *Poales* (Little et al., 2018). Although CslF genes are numerous in maize and rice, CslH is not found in the B73 genome, and the single CslG gene cannot be distinguished as a separate subgroup from CslJ or CslM (**Figure S1**).

Seven of the nine maize CslF genes were expressed at ≥95 reads per 20 M, but five of them were expressed at levels to calculated expression ratios. Four of the five showed primary wall association, but a CslF5 was more strongly expressed during secondary wall formation (**Figure 2C**; **Figure 3C**). The mixed-linkage β-glucans are synthesized during primary wall formation in growing coleoptiles and are largely degraded at the end of elongation (Carpita et al., 2001), but these glucans can continue to be synthesized and persist during secondary wall formation (Vega-Sanchez et al., 2013).

### Genes of Substrate Generation

All eight *sucrose synthase* genes were expressed, with only one associated with secondary wall formation (**Table 1**). Ten families of genes encode enzymes of nucleotide sugar interconversion pathways responsible for synthesis *de novo* of the major neutral and acidic monosaccharides for polysaccharide synthesis (Reiter and Vanzin, 2001; Yin et al., 2011). Apart from duplications in several maize families, genes are generally orthologous based on close phylogenetic relationships and common constitutive or primary wall stage expression patterns (**Figure S2**; **Table S1**). However, at least one member of each family is more highly expressed during secondary wall formation. Although UDP-Ara is synthesized in the pyranose form, a substantial portion of the Ara in cell wall polysaccharides is in the furanose form. Konishi et al. (2007) characterized reversibly glycosylated proteins (RGPs) as UDP-Ara mutases (UAMs) that interconvert UDP-Ara*p* and UDP-Ara*f*. Downregulation of UAM expression results in arabinose deficiencies in both rice (Konishi et al., 2011) and *Arabidopsis* (Rautengarten et al., 2011). Nine maize homologs show high sequence similarity with four of the five *Arabidopsis* RGP (UAM) genes; three of these were highly expressed during secondary wall formation (**Figure S2F**; **Table S1**).

The large multigene family of nucleotide sugar transporters (NSTs) of plant species comprises six subgroups (Handford et al., 2004; Orellana et al., 2016). GONST1 of subgroup III has been defined as a GDP-Man transporter (Baldwin et al., 2001), although GDP-Fuc and UDP-Gal/UDP-Glc are transported by other members of this subgroup (Rautengarten et al., 2014); GONST1 channels substrate to sphingolipid glycosylation and not glucomannan synthesis (Mortimer et al., 2013). Subgroup II includes several UDP-Gal/UDP-Glc transporters (Norambuena et al., 2002), and the UDP-Gal transporter members of subgroup I, or the NST-KT clade, were shown in heterologous systems to have UDP-Rha/UDP-Gal transport activity (Rautengarten et al., 2014). More recently, subgroup V has been shown to contain UDP-GlcA/GalA transporters (Saez-Aguayo et al., 2017) and subgroup VI to contain UDP-Araf transporters (Rautengarten et al., 2017). The maize genome has several NSTs in all six subgroups, including 18 UDP-Gal transporters and UDP-Xyl transporters from subgroup I, five UTRs from subgroup II, and 20 GONST-, NST-, and UTR-like genes in subgroup III (**Figure S3**; **Table S1**). The majority of these were expressed constitutively or predominantly during primary wall formation, but, with the exception of group II, at least one member was highly expressed during secondary wall formation (**Figure S3**; **Table S1**).

### Glucuronoarabinoxylan Synthesis

GAXs are the major noncellulosic glycans in the type II primary walls of grasses. The GAX polysaccharides comprise (1→4)-β-d-xylan backbones with side groups of α-GlcA and α-4-*O*-Me-GlcA linked at the xylosyl *O*-2 position, and Ara*f* residues linked either at the *O*-2 or *O*-3 position depending on species. The grasses show a predominance of substitution of Ara*f* at the *O*-3 xylosyl residue, whereas in noncommelinid species, the Ara*f* residues are at the *O*-2 position (Carpita and Gibeaut, 1993). However, the GAXs of all angiosperm species are capable of adding Ara*f* residues to either or both of the *O*-2 and *O*-3 xylosyl units of the backbone (Carpita, 1996), indicating that genes encoding these transferase activities are expressed in grasses and dicots.

Synthesis of xylan backbones require two distinct members of family GT43, which encode the irregular xylem9 (IRX9) and IRX14 inverting-type xylosyl transferases (Brown et al., 2007; Wu et al., 2009; Smith et al., 2017). Xylan chain synthesis also requires participation of IRX10 and IRX10-like xylan xylosyl transferases of family GT47 subgroup E (Brown et al., 2009; Wu et al., 2010; Zeng et al., 2016). Within this same subgroup is *FRA8*, which is reported to encode a putative xylan-decorating glucuronosyl transferase (Zhong et al., 2005). These two distinct activities within the same GT47 subgroup underscore the need for a more thorough determination of specific function of members of the six subgroups of the GT47 family. Thus far, all are inverting-type glycosyl transferases, i.e., those that convert the α-d- or β-l-nucleotide sugar moiety into a β-d- or α-l-linkage in a polysaccharide, but members of different subgroups use different nucleotide sugars or polymer substrates in transferase reactions (Penning et al., 2009). Arabinosyl and xylosyl side groups are attached by family GT61 inverting-type transferases (Anders et al., 2012; Chiniquy et al., 2012).

**Figure 4:**
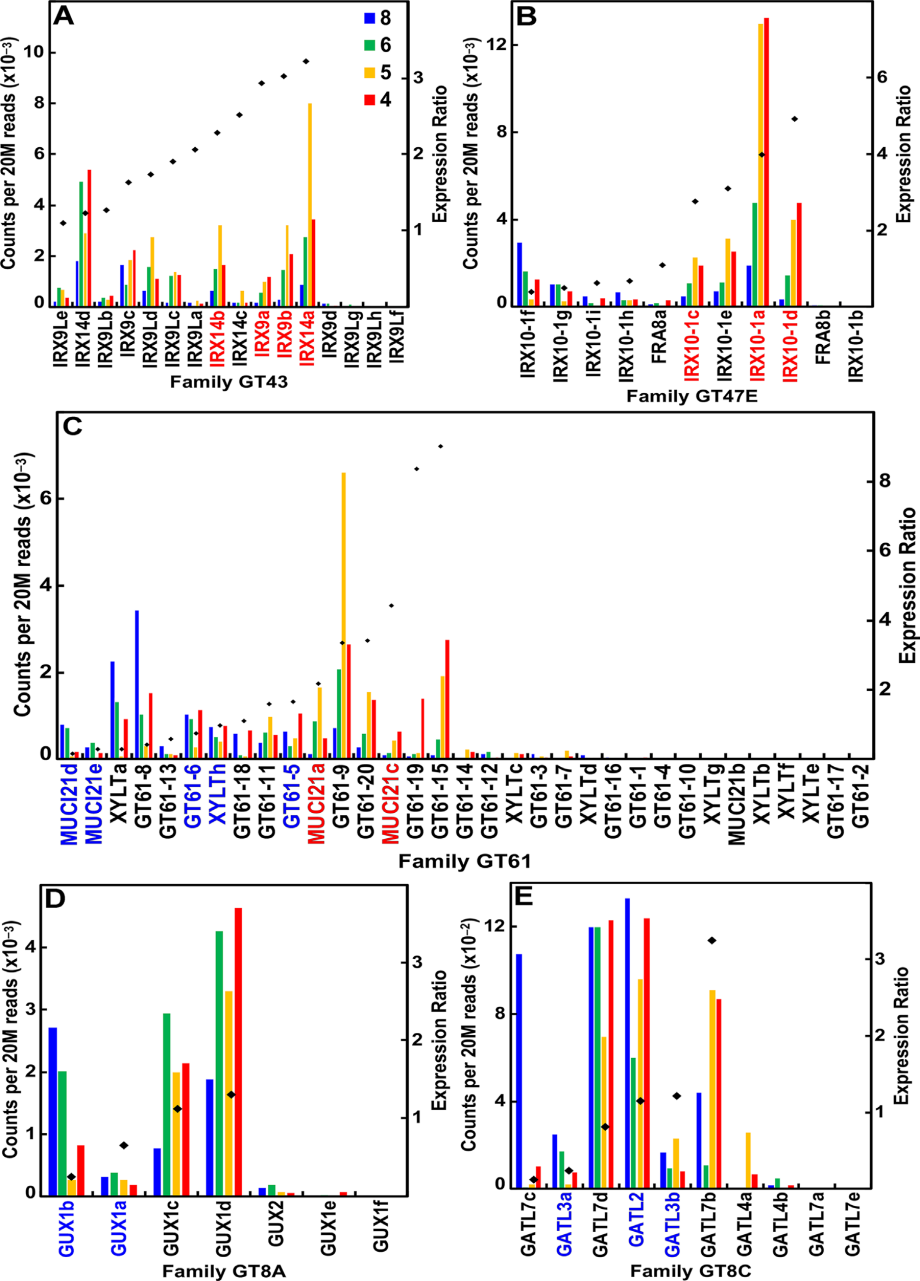
Differential expression of key families of the maize B73 glucuronoarabinoxylan synthesis. Expression ratios and putative *Arabidopsis* orthologs were determined as described in the legend of **Figure 3**. **(A)** Xylan synthases of family GT43. **(B)** Xylan xylosyl transferase of family GT47 subgroup E. **(C)** Arabinosyl and xylosyl transferase of side-chain attachment of family GT61. **(D)** Glucuronosyl transferases (GUXs) of GT8 subgroup A. **(E)** Galacturonosyl transferase-like (GATL) proteins of GT8 subgroup C.

Of the 16 maize *IRX9* and *IRX14* genes, 12 were highly expressed during stem development, 6 of which had expression ratios ≥2 (**Figure 4A**; **Table S1**). Similarly, of the 11 *IRX10* genes in maize, 9 of them were expressed during stem development, and 4 *IRX10-1* genes more highly expressed during secondary wall formation (**Figure 4B**; **Table S1**). The maize family GT61 comprises 33 genes, 6 of which are expressed during secondary cell wall formation (**Figure 4C**; **Table S1**). Glucuronosyl residues are ubiquitous side groups of xylans, the sole sugar substituent of secondary wall xylans. These α-linked GlcA side groups are attached by GT family 8 subgroup A retaining-type glucuronosyl transferases (GUX) by members that display selectivity with respect to the periodicity of GlcA attachment along the xylan backbone (Mortimer et al., 2010). Six of seven maize *GUX* genes were expressed at ≥95 reads per 20 M, with four of them expressed constitutively (**Figure 4D**; **Table S1**). Members of GT8 subgroup C of *galacturonosyl transferase-like* (*GATL*) genes function to initiate GX synthesis through participation in the synthesis of a tetrasaccharide primer sequence (Lee et al., 2007). Maize GATL2 is homologous with the *Arabidopsis* *PARVUS* gene, which was constitutively expressed, whereas *GATL7b* showed high secondary wall expression (**Figure 4E**; **Table S1**).

In addition to the sugar side groups, xylans can have high degrees of acetylation, and the large family of *trichome-birefringence-like* (*TBL*) and *TBL-like* genes encode acetyl transferase enzymes, at least some of which are involved specifically in acetylation of xylans (Gille and Pauly, 2012; Gao et al., 2017). The maize *TBL* family comprises 56 genes in five subgroups, with 7 additional genes related to *reduced wall O-acetylation* (*RWA*) and two homologs to *Arabidopsis* *xyloglucan acetyl transferase9* (*AXY9*); a majority of these acetyl transferases were expressed constitutively or higher during primary wall formation (**Table S1**), but several exhibited particularly high expression during secondary wall formation (**Figure S4**). One of the hallmarks of the grass cell wall is the autofluorescence of the primary wall from a phenylpropanoid network integrated with xylans. Ferulic acid and *p*-coumaric acid are known to be extended from arabinosyl residues of GAX, and the BAHD family contains CoA-dependent transferases thought to participate in addition of these hydroxycinnamic acids (Rautengarten et al., 2012; Molinari et al., 2013). The maize BAHD family numbers 12 genes, of which 3 are highly expressed during secondary wall formation (**Figure S5**; **Table S1**).

### Xyloglucan Synthesis

The principal cross-linking glycans of dicots and noncommelinid monocots are XyGs, whose fundamental structure is a (1→4)-β-d-glucan backbone branched at the *O*-6 by α-d-Xyl residues (Carpita and Gibeaut, 1993). In most angiosperms, including *Arabidopsis*, three consecutive glucosyl residues of every four in the backbone are subtended by Xyl residues, and the two Xyl residues closer to the reducing end of the backbone can be substituted further at the *O*-2 of one or both positions with β-d-Gal residues (Scheller and Ulvskov, 2010). If a Gal residue subtends the Xyl residue closest to the reducing end, an α-l-Fuc is likely to be added to the Gal *O*-2 position. The glucan backbones of XyGs of grasses are irregularly branched with one or two Xyl residues, and these contain an occasional Gal residue (Carpita, 1996).

From heterologous expression experiments, genes of the CslC subgroup likely encode the XyG glucan backbone synthases (Cocuron et al., 2007), and genes of the GT34 family encode the XyG xylosyl transferases (XXTs) (Cavalier et al., 2008; Zabotina et al., 2012). The *mur3* mutation was traced to a gene encoding a xyloglucan galactosyl transferase (GalT) in family GT47 subgroup A, whose product decorates the xylosyl residue closest to the reducing end of the oligomer unit (Madson et al., 2003), and the subgroup A homolog GalT decorates the “middle” xylosyl residue (Kong et al., 2015). When a Gal is added to the first Xyl residue, it becomes a possible substrate for a family GT43 transferase that fucosylates it (Vanzin et al., 2002).

**Figure 5:**
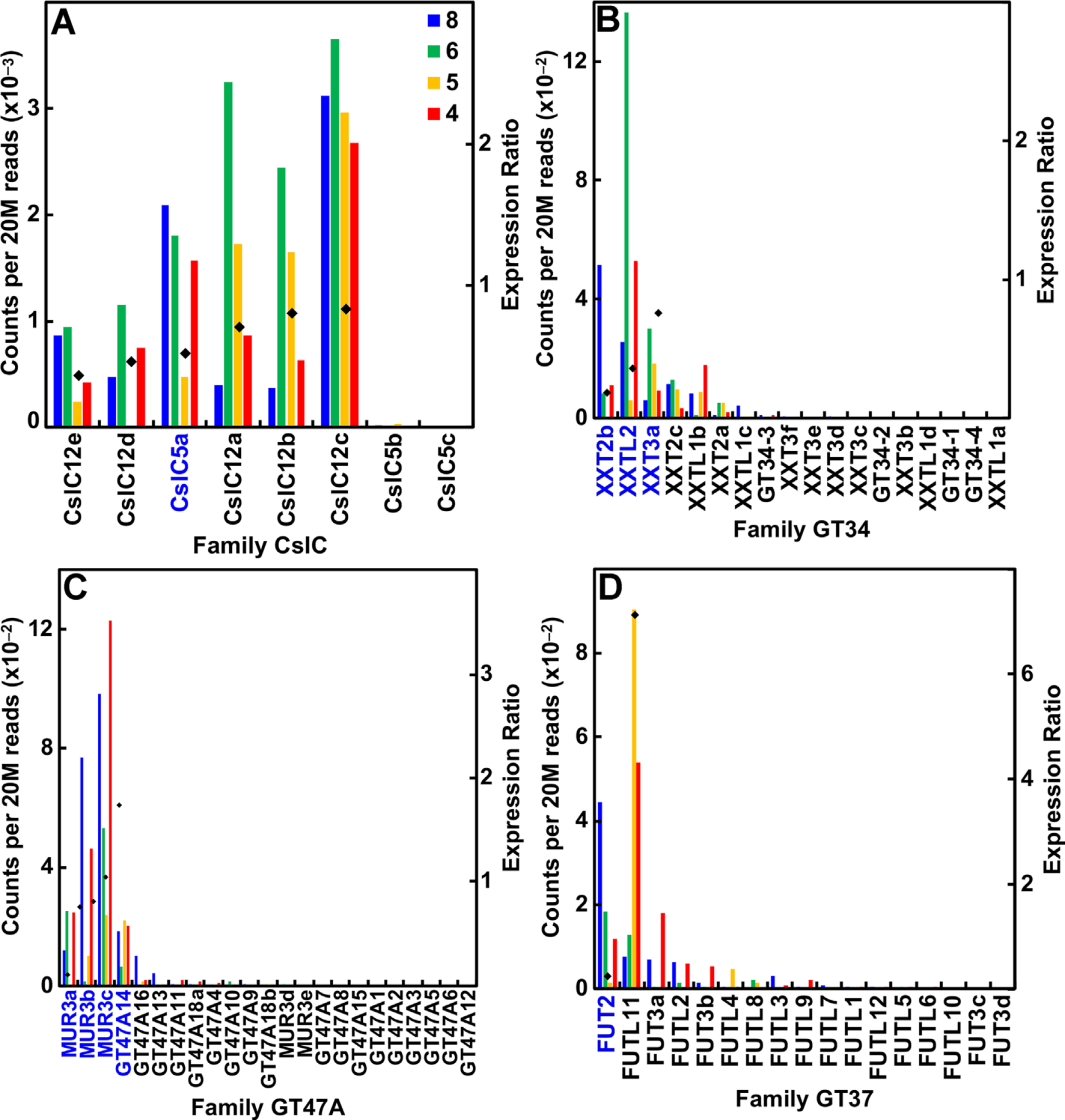
Differential expression of key families of the maize B73 XyG synthesis. Expression ratios and putative *Arabidopsis* orthologs were determined as described in the legend of **Figure 3**. **(A)** XyG backbone synthases of family CslC. **(B)** Xyloglucan xylosyl transferases of family GT34. **(C)** Galactosyl transferases of family GT47 subgroup A. **(D)** Fucosyl transferases of family GT37.

Despite small amounts of the truncated form of XyGs that accumulate in the walls of maize and other grasses, these species have the capacity to synthesize a fucosylated XyG. The maize genome contains eight CslC genes and expressed six of them (**Figure 5A**; **Table S1**). Expression of all six was associated with primary wall formation. Only one of these genes, *CslC5a*, had an expression profile and sufficient sequence similarity with *Arabidopsis* to be considered a potential ortholog. The maize genomes possess 17 Family GT34 *XXT* and *XXT-like* genes (**Figure 5B**; **Table S1**). The more highly expressed members of the family had expression ratios that indicated association with primary wall XyG synthesis. The maize genome also has 22 members of family GT47 subgroup A that encodes the XyG-specific Gal transferases, four of which were highly expressed at early stages of stem development. Three of them are putatively orthologous to *MUR3* (**Figure 5C**; **Table S1**). Despite the lack of detectable fucosylation of XyGs of grasses, maize also has 17 GT37 *fucosyl transferases* (*FUTs*); four of these were expressed at ≥500 reads per 20 M, one (*FUT2*) during primary cell wall growth stages and one (*FUT11*) with strong expression during secondary wall formation (**Figure 5D**; **Table S1**).

### Pectin Synthesis

Although the type II cell walls of commelinid monocots generally have limited amounts of pectic polysaccharides, they are found transiently in higher abundance in some developmental contexts, such as endosperm development (Chateigner-Boutin et al., 2014; Zhang et al., 2016). The pectin matrix consists primarily of two kinds of pectic polysaccharide backbones of (1→4)-α-d-homogalacturonan (HG) and repeating units of the *O*-2-α-d-Rha-(1→4)-α-d-Gal disaccharide in rhamnogalacturonan-I (RG-I) (Caffall and Mohnen, 2009). Some HGs are branched with Xyl residues to form Xyl-HGs or possess clusters of four complex oligosaccharides to form RG-II, a polysaccharide that forms boron di-diester crosslinks. Branched (1→5)-α-l-arabinans and type I (1→4)-α-d-galactans with appendant Ara residues are attached typically to the Rha *O*-4 position of RG-I.

The maize genome contains numerous genes associated with the synthesis of pectins. The GT8 subgroup D retaining-type *galacturonosyl transferase* (*GAUT*) family comprises 23 genes, and all of them were expressed at ≥95 reads per 20 M throughout stem development (**Figure S6A**; **Table S1**). The vast majority of them were expressed at primary wall or transitional stages, but two nonorthologous genes, *GAUT11b* and *GAUT11c*, displayed higher relative expression during secondary wall synthesis. Pectin RG-I synthesis requires a rhamnosyl transferase (RRT1) from family GT106 (Takenaka et al., 2018). From gene expression related to seed mucilage RG-I synthesis, the *Arabidopsis* GAUT11 is the prime candidate for the GalA transferase associated with RG-I synthesis (Voiniciuc et al., 2018), with a close homolog also found in maize (**Table S1**). The maize genome also contains a large family of GT106 genes, including three of four RRTs that are expressed mostly constitutively, but with RRT1b more highly expressed during secondary wall formation (**Figure S6B**). A related subgroup of GT106 are the *pectin arabinogalactan synthesis-related* (PAGR; Stonebloom et al., 2016) genes, and mannan synthesis-related transferase (MSR; Wang et al., 2013) genes. Seven of the nine maize PAGR genes were expressed mostly during primary wall formation, but PAGR-L1 showed higher expression during transitional stages of development (**Figure S6C**). All six MSR genes were constitutively expressed (**Table S1**).

The maize genome contains a vast number of genes that encode enzymes of pectin depolymerization. *Polygalacturonase* (*PGase*) genes comprise six subgroups with high expression of members in all of them except subgroups E and F (**Figure S7**; **Table S1**). None is expressed specifically during secondary wall formation. Maize also has four *RG-I lyases* (*RGIL*s), with only *RGIL1* significantly expressed (**Figure S7F**).

### AGPs and Other GPI-Anchored Proteins

GPI-anchored peptidoglycans, such as type II arabinogalactan proteins (AGPs), with their highly branched (1→3)-, (1→6)-, and (1→3, 1→6)-β-d-galactan chains and Ara side groups, are found in small amounts in primary cell walls (Showalter, 1993; Borner et al., 2003; Johnson et al., 2003). Two, *FLA2c* and *FLA11*, of seven AGP/fasciclin genes showed relatively higher secondary wall expression (**Figure S8A**; **Table S1**). Family GT31 represents a large family of six subgroups and includes GalTs that are predicted to form the (1→3)-β- and (1→6)-β-linked galactan chains of type II AGPs. Most of the GT31 genes are expressed during primary wall formation, but one member of GT31 subgroup A, *GT31A3*, and two members of subgroup F, *GT31F4* and *GT31F5*, exhibited predominantly secondary wall expression (**Figure S8B**; **Table S1**).

Two notable families of GPI-anchored proteins discovered in *Arabidopsis* are *skewed growth* (*SKU*) proteins, mutations in which result in abnormal growth symmetry (Rutherford and Masson, 1996; Sedbrook et al., 2002), and COBRA proteins involved in determining the direction of wall expansion and patterning of cellulose in primary walls (Schindelman et al., 2001) and cellulose content and tensile strength of the floral stem (Brown et al., 2005). Maize *SKU* genes number 13, with seven expressed predominantly during primary wall formation (**Figure S9**; **Table S1**). Nine *COBRA* genes comprise the maize family, with three expressed more or less constitutively and *COBL4b* expressed during secondary wall formation (**Figure S9**; **Table S1**).

### Expansins and Endotransglucosylase/Hydrolases

The cell wall is residence to hundreds of enzymes, such as expansins and transglucosylases, polysaccharide hydrolases and lyases, oxido-reductases, and proteases, which function in wall remodeling and metabolism (Boudart et al., 2005; Hervé et al., 2016), and maize expresses many of these during both primary and secondary wall stages of growth (**Table S1**). Expansins and the GH16 family of xyloglucan endotransglucosylase/hydrolases (XTHs) are implicated in cellulose microfibril separation during growth and the rejoining of XyGs to maintain tensile strength, respectively (Cosgrove, 2000; Rose et al., 2002). The maize genome contains over 50 *α-Expansin* (*α-Exp*), *α-Expansin-like* (*α-Exp-like*), and *β-Expansin* (*β-Exp*) genes, with most expressed during early growth except for an *α-Exp8a* and an *α-ExpL2c* more highly expressed during secondary wall formation (**Figure S10**; **Table S1**). Similarly, 30 maize *XTH* genes form three subgroups, with the majority of them expressed during elongation and primary wall stages of growth, but three *XTHB* genes and one *XTHC* gene were more highly expressed during secondary wall formation (**Figure S11**; **Table S1**).

### Monolignol and Lignin Synthesis

**Figure 6:**
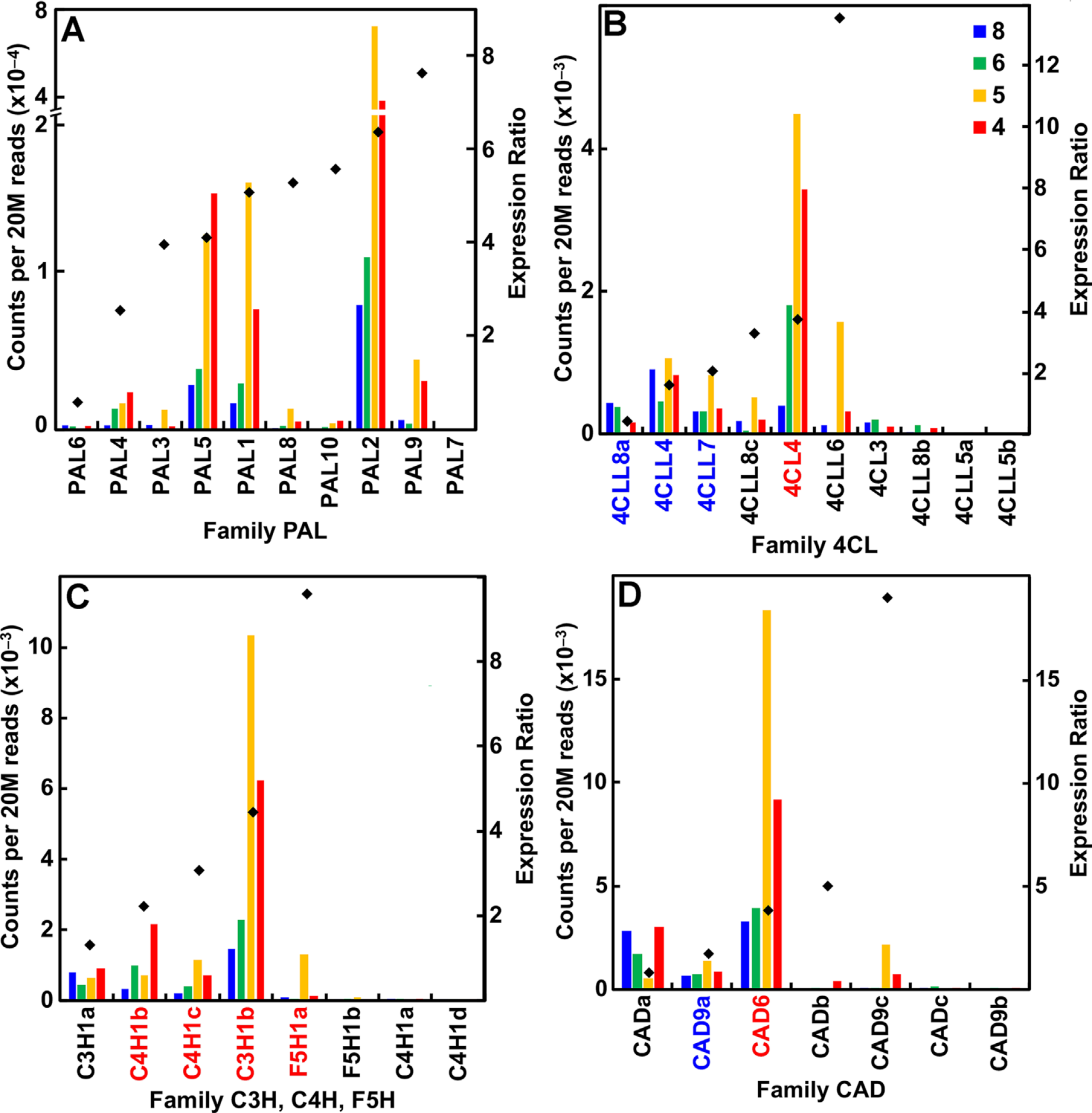
Differential expression of key families of the maize B73 monolignol synthesis. Expression ratios and putative *Arabidopsis* orthologs were determined as described in the legend of **Figure 3**. **(A)** Family PAL, phenylalanine ammonia lyases. **(B)** Family 4CL, 4-coumarate CoA ligases. **(C)** Families C3H (coumarate-3-hydroxylases), C4H (cinnamate-4-hydroxylases), and F5H (ferulate-5-hydroxylases). **(D)** Family CAD, cinnamyl alcohol dehydrogenases.

A major distinction of the type II primary cell walls of grasses is the presence of a phenylpropanoid network (Carpita, 1996). Several members in each family of genes that encode the enzymes of monolignol synthesis were expressed during primary wall synthesis, even though most of the genes were upregulated in older internodes. All but one of the *phenylalanine/tyrosine ammonia lyase* (*PAL*) genes were expressed in secondary cell-wall-forming tissues (**Figure 6A**; **Table S1**). In contrast, expression of all other genes of monolignol formation, such as *4-coumarate CoA ligase*, *cinnamyl alcohol dehydrogenase*, *hydroxycinnamoyl-CoA shikimate/quinate hydroxycinnamoyl* *transferase*, *cinnamyl CoA reductase* (*CCR*), and *caffeoyl-coenzyme A 3-O-methyltransferase* (*CCoAOMT*), were expressed during primary wall formation and secondary wall formation in roughly equal numbers (**Figures 6B–D**; **Table S1**). Peroxidases are encoded by a huge family of 86 genes in seven subgroups and *Laccases* numbered 24 genes (**Table S1**). Although expression ratios showed genes with strong elongation-specific expression or secondary wall expression, ratios were widely clustered between 2 and 12, indicating more complex patterns of expression.

### Other Wall-Related Proteins

Apart from extensin domains within some of the AGP proteoglycans, the classic Ser-Hyp_4_-type extensin proteins are in low abundance in the maize genome. Nevertheless, we identified several genes related to their synthesis and glycosylation. For example, all but 1 of the 10 prolyl-4-hydroxylases that synthesize Hyp were expressed, and all of these during primary wall formation. Of the 23 genes that encode family GT77 arabinosyl transferases, which attach the Ara side groups of extensin, 12 were expressed at ≥95 reads per 20 M, mostly during primary wall stages, but 1, *reduced residual arabinose1b* (*RRA1b*; Egelund et al., 2007), was more strongly expressed during secondary wall formation (**Table S1**).

## Discussion

The progenitor species of commelinid monocots diverged from dicotyledonous and other monocotyledonous species about 120 million years ago (Hertweck et al., 2015) to species that made cell walls with mostly GAX as the cross-linking glycan, and with much less XyG and pectin than their common eudicot ancestors (Smith and Harris, 1999). The feruloylation of the Ara residues in the commelinid species and the initiation of phenylpropanoid networks in the primary walls of all commelinids gave characteristic strong autofluorescence in UV light (Rudall and Caddick, 1994). The Order Poales split from its closest commelinid relatives about 65 million years ago (Hertweck et al., 2015), with expansion of CslF and CslH subgroups that encode the synthases of the mixed-linkage β-glucans (Burton et al., 2006; Doblin et al., 2009).

Despite the evolution of commelinid species with cell walls distinct from dicots and noncommelinid monocots, we show here that their genomes retained the capacity to make the cell wall polysaccharides of all angiosperm species. In addition, families of genes for synthesis of polysaccharides uncharacteristic of the two wall types are expressed in the expected developmental context of primary or secondary wall. Key questions are if the transcripts expressed become translated into protein, and if so, are these uncharacteristic polysaccharides actually made? Our recent work in comparative glycome and proteome analysis indicated that *Arabidopsis* and maize, representative species with type I and type II cell walls, respectively, synthesize and accumulate, in the Golgi, polysaccharides that are not found in abundance in their cell walls (Okekeogbu et al., 2019). Maize Golgi had a higher proportion of XyG to GAX, and *Arabidopsis* had a higher proportion of GAX to XyG in their respective Golgi. Three possible explanations were considered for these findings: (1) that polysaccharides that accumulate to higher abundances in Golgi might result from lower trafficking rates compared to higher rates for material trafficked to the wall, (2) that the lower trafficking rates might reflect diversion of a subset of polysaccharides to lytic compartments instead of the cell wall, or (3) that all polysaccharides are trafficked to the wall, but polysaccharides uncharacteristic of wall type are digested extracellularly and, therefore, fail to accumulate. As the *trans*-Golgi network/early endosome compartment plays a central role in post-Golgi synthesis, sorting, and packaging of polysaccharide and protein cargoes destined for the wall (Kang et al., 2011; Rosquete et al., 2018), we suggest that this compartment might be the site of discrimination.

The broader question of evolutionary significance is why a species would place a large metabolic investment in polymers that never accumulate in the wall. However, retaining the capacity to make alternative polysaccharides if mutations occur could be a selective advantage. If mutations occur in synthases of the characteristic polysaccharide, viability is not jeopardized if an alternative polysaccharide can be made. Such plasticity in wall composition is illustrated by the mutations that introduce severe alterations in polysaccharide structure or abundance yet plants maintain near normal growth and development. Among the starkest examples are the *Arabidopsis* *xxt1/xxt2* double mutant that completely lacks XyG in the wall, but despite a slight lowering of tensile strength, plant structure is remarkably unchanged from wild type (Cavalier et al., 2008; Zabotina et al., 2012), and the survival of cells in liquid culture in the near absence of cellulose induced by a potent cellulose synthesis inhibitor regardless of wall type (Shedletzky et al., 1990; Shedletzky et al., 1992).

## Conclusions

While it was tempting to hypothesize that the evolution of a completely distinct type of cell wall resulted in a drastic change in the gene families that encode its synthesis machinery, expression of specific gene family members is not the basis for the difference. Although a few examples exist of the divergence of certain subgroups of families unique to grass species, the vast majority are populated with members in all angiosperms. Divergence within a subgroup gives evidence of neofunctionalization during speciation, but it is equally evident that grass species express large numbers of genes of these subfamilies that encode polysaccharides that accumulate in the Golgi but never traffic to or assemble in the wall. Thus, the type of cell wall made is established by post-Golgi mechanisms that remain to be determined. The capacity to make in any species the entire repertory of cell wall polysaccharides widens the spectrum of design properties for cell walls as materials with emergent properties.

## Data Availability Statement

An improved annotation of maize cell wall protein families is searchable at Maize GDB (https://www.maizegdb.org/gbrowse/maize_v2test?l=CellWallGenes;l=Gene_models;q=Chr1:2650000.2699999). This resource is also downloadable at (http://cellwall.genomics.purdue.edu). RNA-seq data are available at https://www.ncbi.nlm.nih.gov/sra/PRJNA522448 (datasets: SRX5387736, SRX5387731, SRX5387711, SRX5387715).

## Author Contributions

BP, MM, and NC designed the research. BP carried out the experiments with assistance from MM and NC, and BP, MM, and NC analyzed the results and wrote the manuscript.

## Funding

The work on cell-wall gene annotations was supported by the Center for Direct Catalytic Conversion of Biomass to Biofuels, an Energy Frontier Research Center funded by the US Department of Energy, Office of Science, Office of Basic Energy Sciences (grant no. DE-SC0000997), and the expression analyses were supported by the US Department of Energy Feedstock Genomics Program, Office of Biological and Environmental Research, Office of Science (grant no. DE-FOA-0000598). Mention of trade names or commercial products in this publication is solely for the purpose of providing specific information and does not imply recommendation or endorsement by the US Department of Agriculture. USDA is an equal opportunity provider and employer.

## Conflict of Interest

The authors declare that the research was conducted in the absence of any commercial or financial relationships that could be construed as a potential conflict of interest.
