# Supplementary material for: Evolution of the Cell Wall Gene Families of Grasses
Source: Front Plant Sci. 2019 Oct 4;10:1205. doi: 10.3389/fpls.2019.01205 (PMC6805987; doi:10.3389/fpls.2019.01205)
Supplement: Supplementary file 1 [file DataSheet_1.pdf]

Supplementary Material: Data sheet 1

Evolution of the cell wall genome of grasses

Bryan W. Penning[1], Maureen C. McCann[2,4], Nicholas C. Carpita[2,3,4*]

[1]USDA-ARS, Corn, Soybean and Wheat Quality Research, 1680 Madison Avenue, Wooster, OH 44691 USA

[2]Department of Biological Sciences, Purdue University, 915 West State Street, West Lafayette, IN 47907 USA

[3]Department of Botany & Plant Pathology, Purdue University, 915 West State Street, West Lafayette, IN 47907 USA

[4]Purdue Center for Plant Biology, West Lafayette, IN 47907 USA

*for correspondence:

Nicholas C. Carpita
Department of Botany & Plant Pathology
915 West State Street
Purdue University
West Lafayette, IN 47907
Tel. (765) 494-4653
E-mail: carpita@purdue.edu

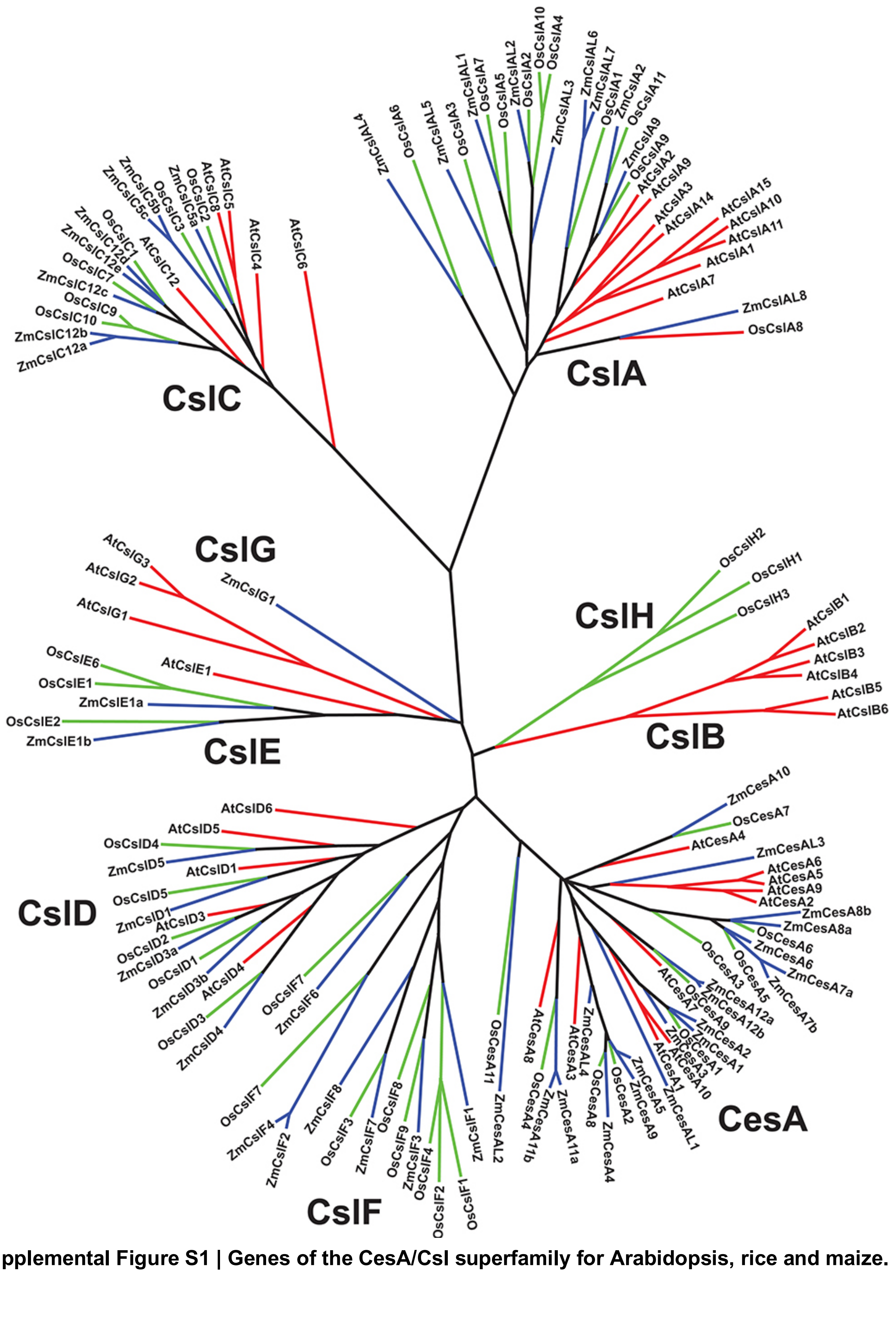

Supplemental Figure S1 | Genes of the CesA/CsI superfamily for Arabidopsis, rice and maize.

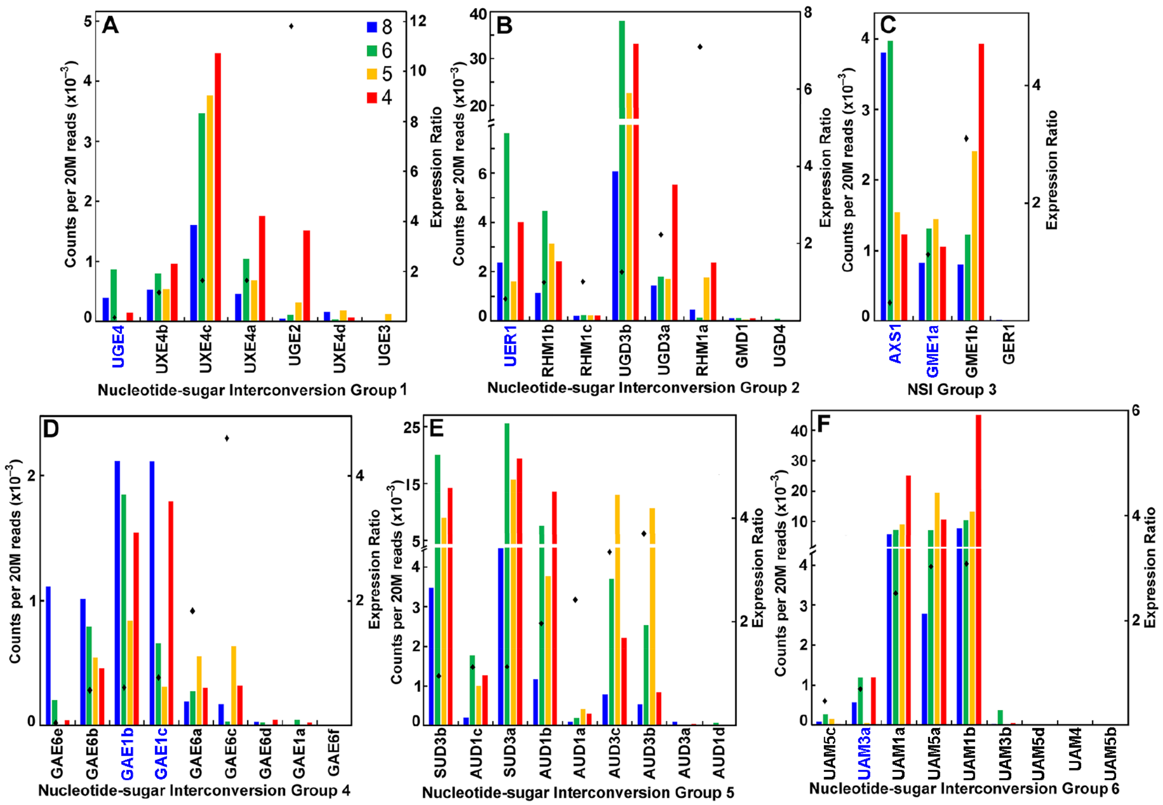

Supplemental Figure S2 | Differential expression of families of the maize B73 nucleotide-sugar interconversion. Expression ratio is calculated as the sum of the reads of Internodes 4 and 5 divided by the sum of reads of Internodes 6 and 8. The expression of maize family member genes is ordered by their ratio of expression in secondary cell wall forming tissue to elongating tissue (black diamonds). Putative orthologs of Arabidopsis also involved in primary wall (blue) and secondary wall (red) synthesis are shown. **(A)** Family UXE, UDP-D-xylose 4-epimerases; and Family UGE, UDP-D-glucose 4-epimerases. **(B)** Family UER, UDP-4-keto-6-deoxy-D-glucose 3,5-epimerase-4-reductases; Family GMD, GDP-D-mannose 4,6-dehydratases; Family RHM, UDP-L-rhamnose synthases; and Family UGD, UDP-D-glucose dehydrogenases. **(C)** Family AXS, UDP-D-apiose/UDP-D-xylose synthases; Family GME, GDP-D-mannose 3,5-epimerase; and Family GER, GDP-4-keto-6-deoxy-D-mannose 3,5-epimerase-4-reductases. **(D)** Family GAE, UDP-D-glucuronate 4-epimerases. **(E)** Family AUD, membrane-anchored UDP-D-glucuronate decarboxylase; and SUD, soluble UDP-D-glucuronate decarboxylase. **(F)** Family GT75, RGP (UAM), Reversibly glycosylated proteins (UDP-L-arabinose mutases).

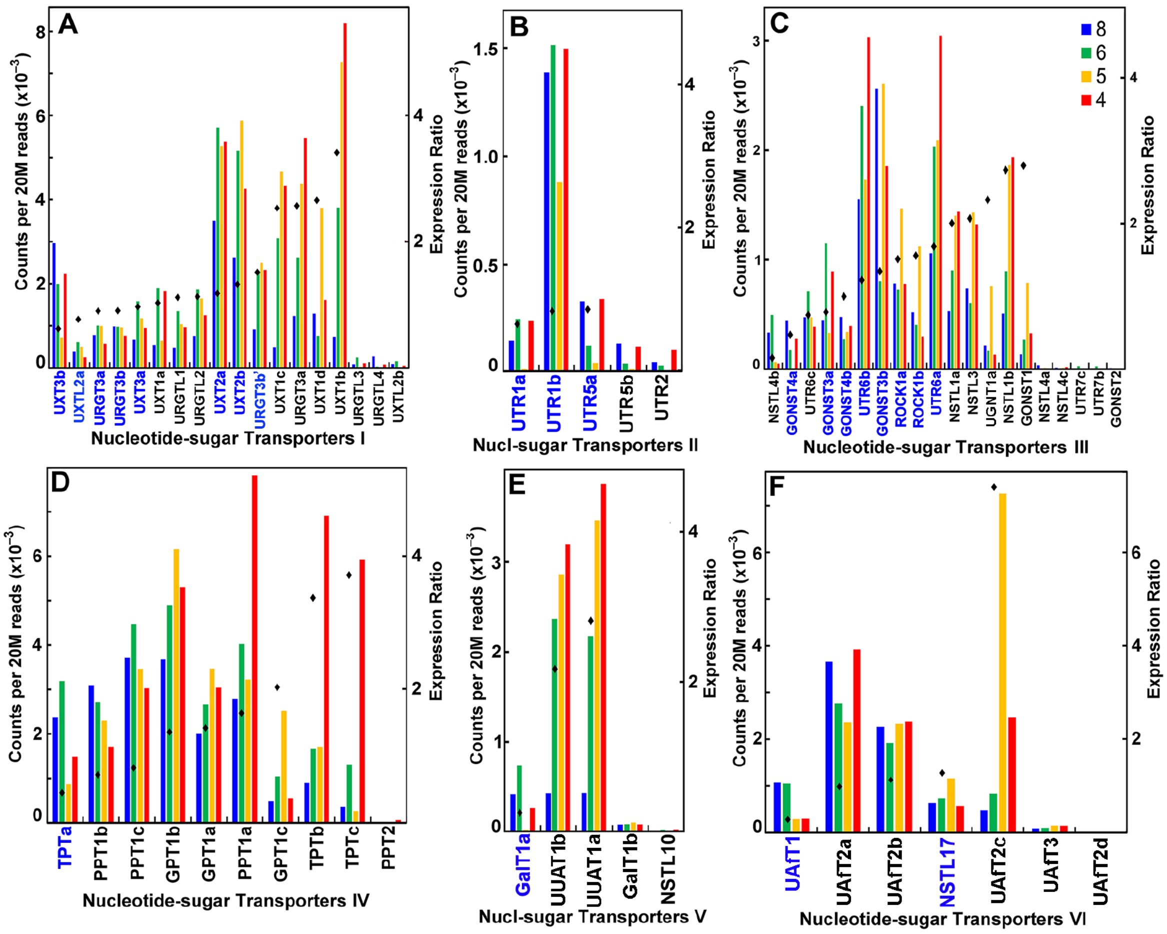

Supplemental Figure S3 | Differential expression of families of the maize B73 nucleotide-sugar transport. Expression ratios and putative Arabidopsis orthologs were determined as described in the legend of Figure S2. Putative orthologs of Arabidopsis also involved in primary wall are shown in blue. **(A)** Group I transporters. **(B)** Group II transporters. **(C)** Group III transporters. **(D)** Group IV transporters. **(E)** Group V transporters. **(F)** Group VI transporters.

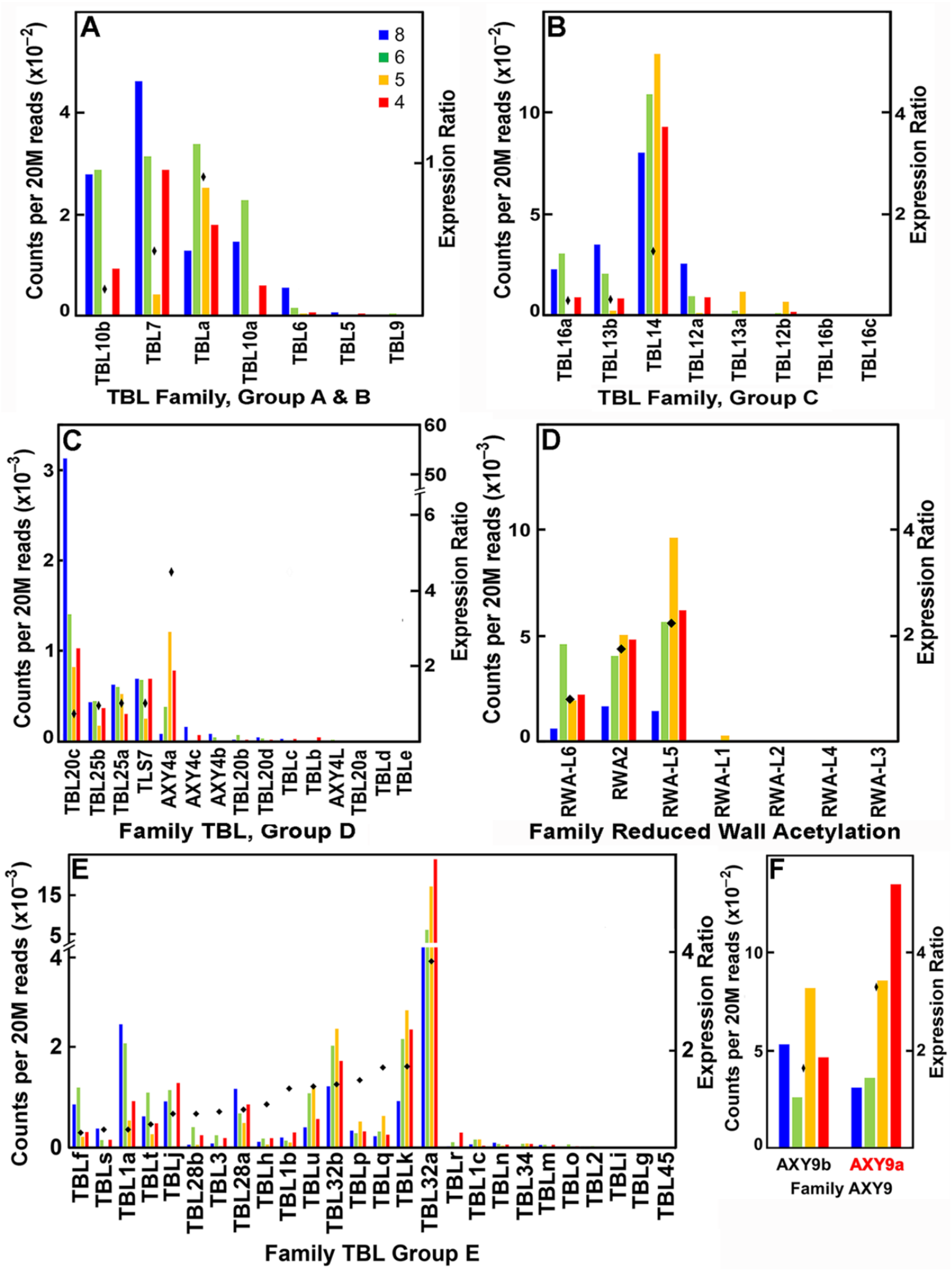

Supplemental Figure S4 | Differential expression of families of the maize B73 glucuronoarabinoxylan modification. Expression ratios and putative Arabidopsis orthologs were determined as described in the legend of Figure S2. (**A**) Family Trichome Birefringence-like (TBL) acetyl-transferases, Groups A and B. **(B)** Family TBL, Group C. **(C)** Family TBL, Group D. (**D**) The Reduced Wall Acetylation (RWA) Family. **(E)** TBL, Group E. **(F)** Family Xyloglucan Acetyltransferase9.

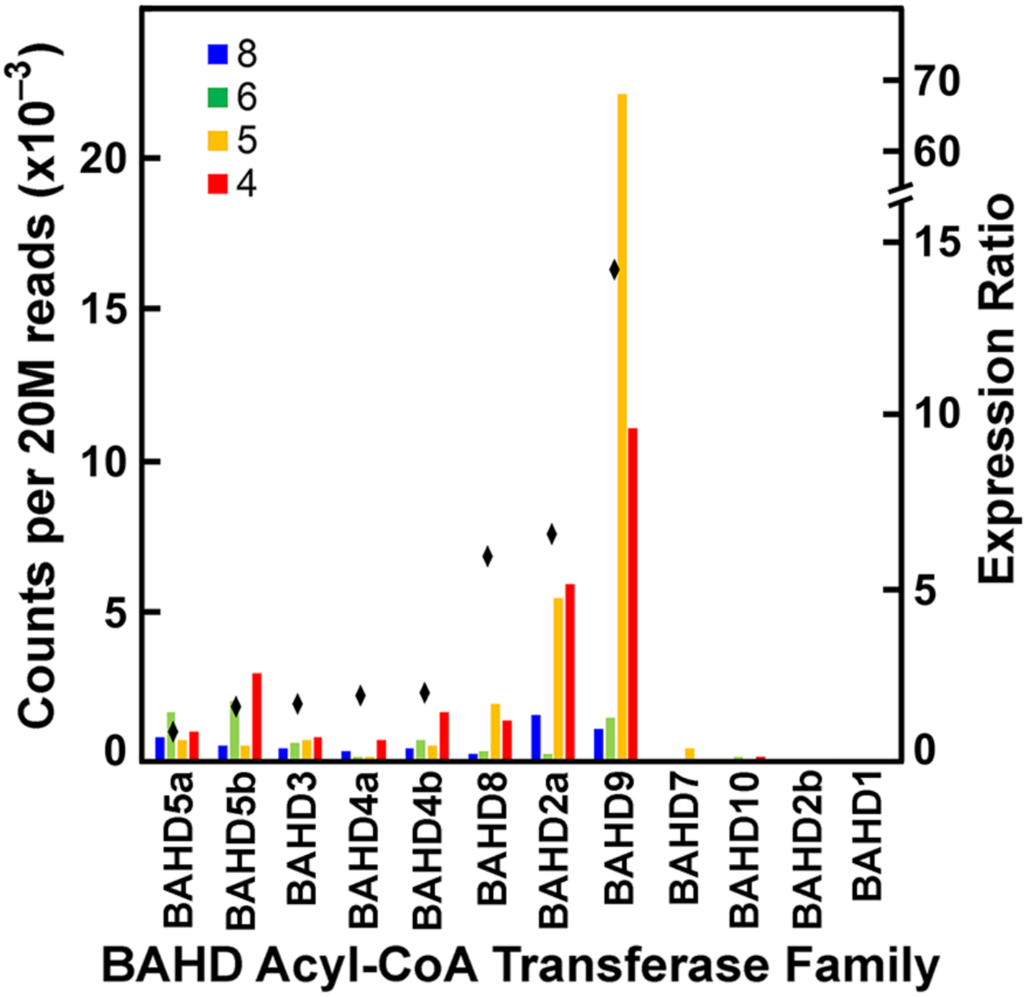

Supplemental Figure S5 | Differential expression of families of the maize B73 BAHD family of acyl-CoA transferases. Expression ratios were determined as described in the legend of Figure S2. No orthologs of Arabidopsis were found.

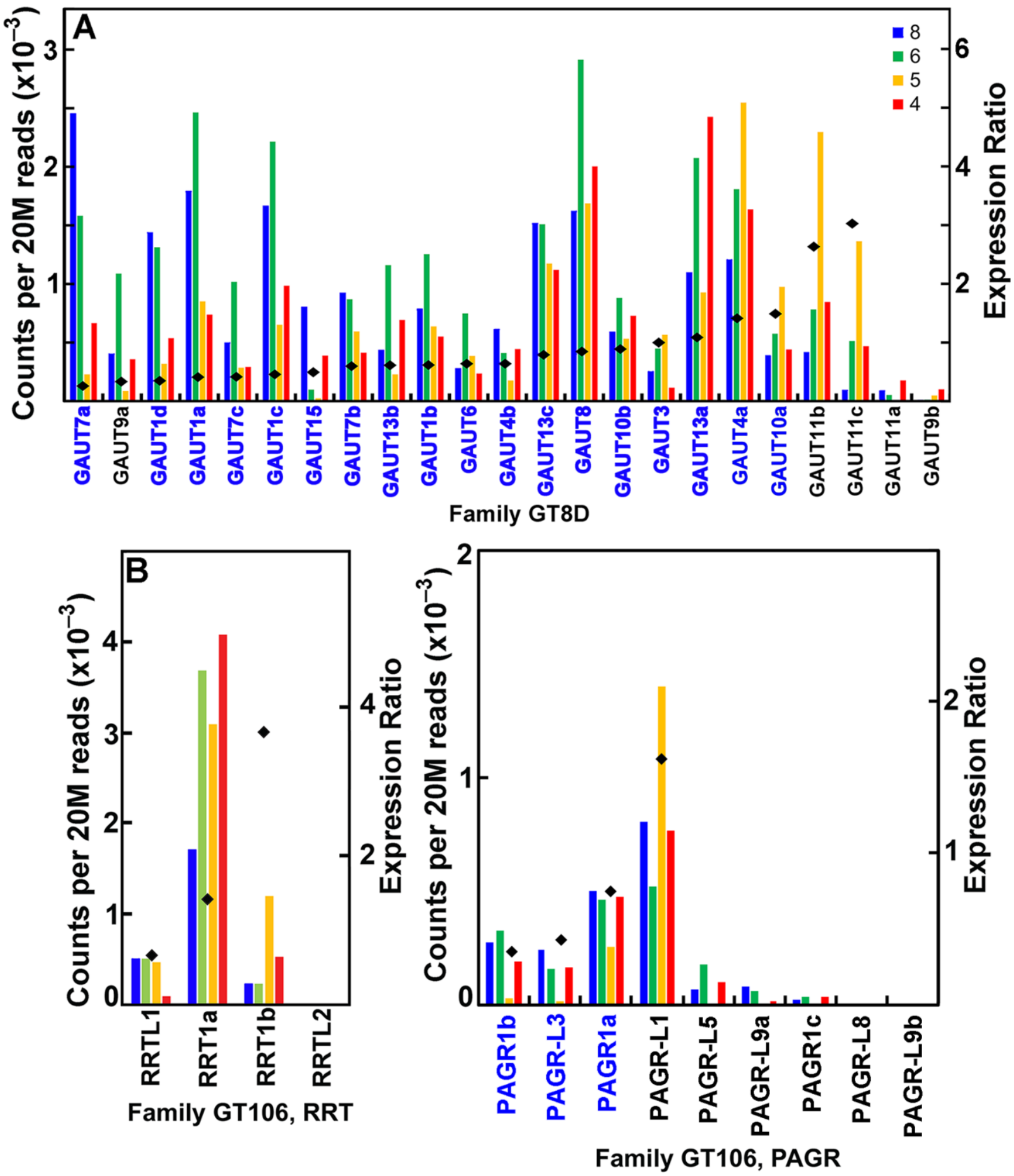

Supplemental Figure S6 | Differential expression of families of the maize B73 polygalacturonan and RG-I synthesis. Expression ratios and putative Arabidopsis orthologs were determined as described in the legend of Figure S2. **(A)** Family GT8 subgroup D, Galacturonosyl transferases **(B)** Family GT106 retaining glycosyl transferases, rhamnosyl transferases (RRTs). **(C)** Family GT106 retaining glycosyl transferases of the Pectin Arabinogalactan-related transferases (PAGRs).

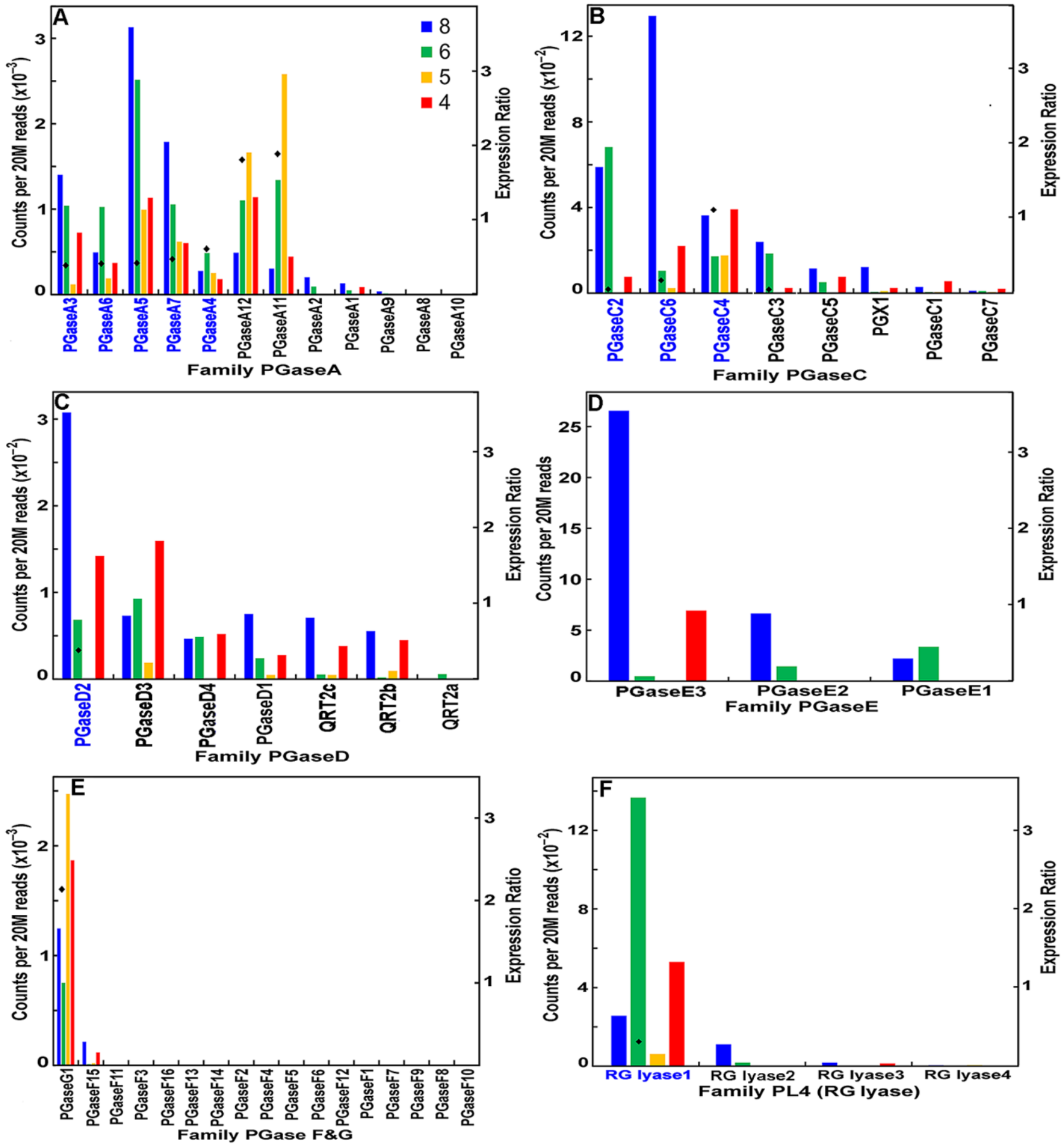

Supplemental Figure S7 | Differential expression of families of the maize B73 polygalacturonase superfamily and Family RG lyase. Expression ratios and putative Arabidopsis orthologs were determined as described in the legend of Figure S2. **(A)** Family polygalacturonase, subgroup A. **(B)** Family polygalacturonase, subgroup C. **(C)** Family polygalacturonase, subgroup D. **(D)** Family polygalacturonase, subgroup E. **(E)** Family polygalacturonase, subgroups F and G. **(F)** family PL4, rhamnogalacturonan-I lyases (RGILs).

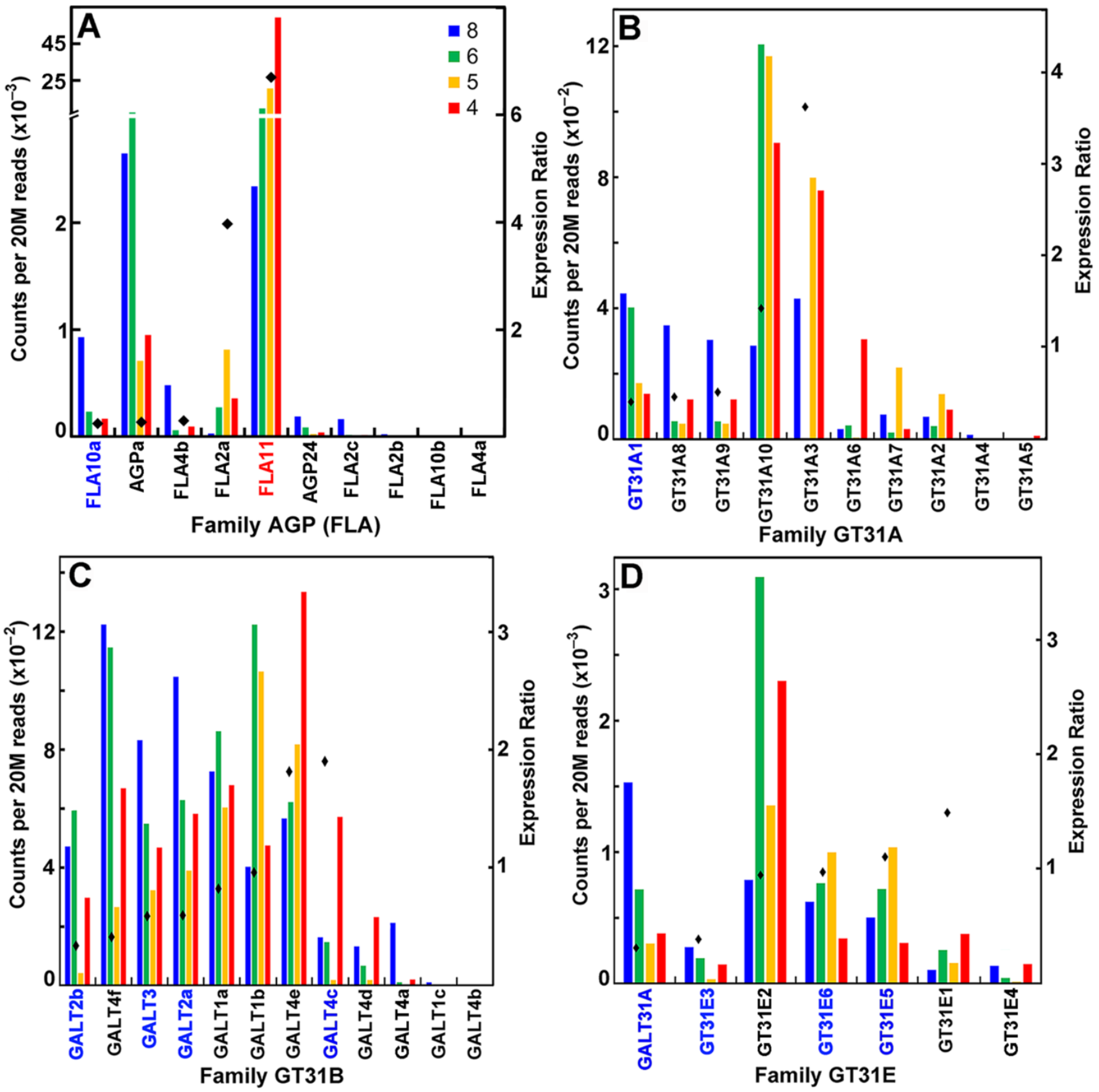

Supplemental Figure S8. Differential expression of families of the maize B73 Family AGP/FLA and Family GT31. Expression ratios and putative Arabidopsis orthologs were determined as described in the legend of Figure S2. **(A)** Family AGP/FLA. **(B)** GT31 subgroup A. **(C)** Family GT31, subgroup B, galactosyl transferases. **(D)** Family GT31, subgroup E.

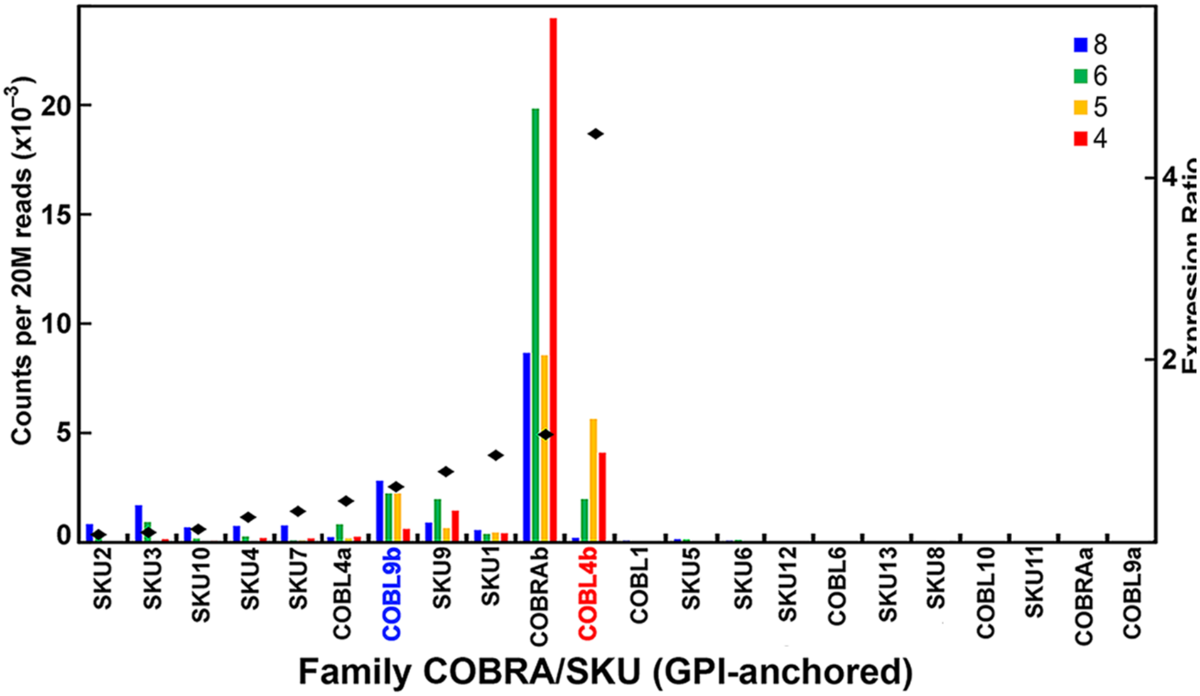

Supplemental Figure S9. Differential expression of families of the maize B73 Family SKU and COBRA GPI-anchored proteins. Expression ratios and putative Arabidopsis orthologs were determined as described in the legend of Figure S2.

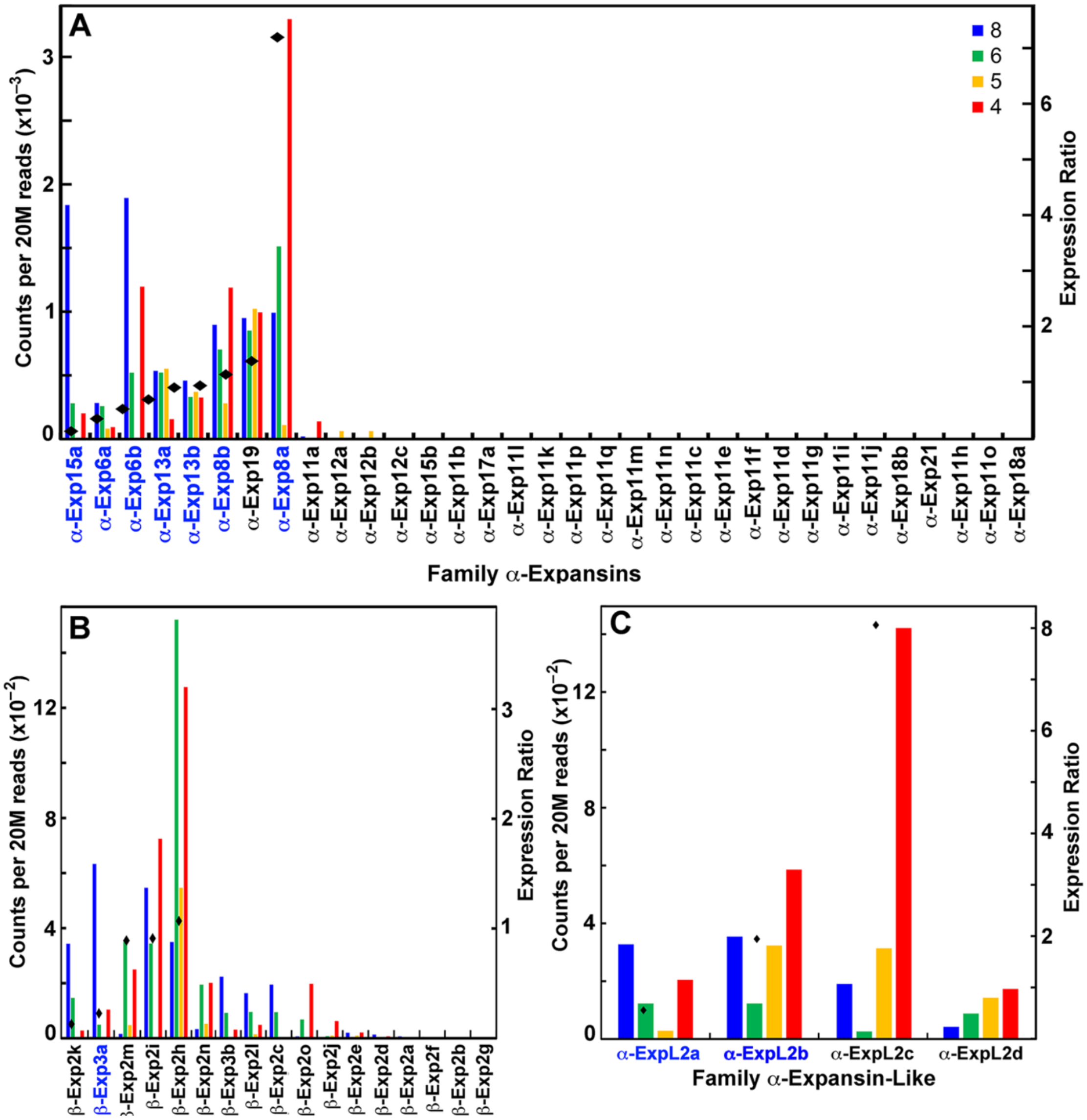

Supplemental Figure S10. Differential expression of families of the maize B73 expansin family. Expression ratios and putative Arabidopsis orthologs were determined as described in the legend of Figure S2. **(A)** Family α-Expansins. **(B)** Family β-Expansins. **(C)** Family α-Expansin-like.

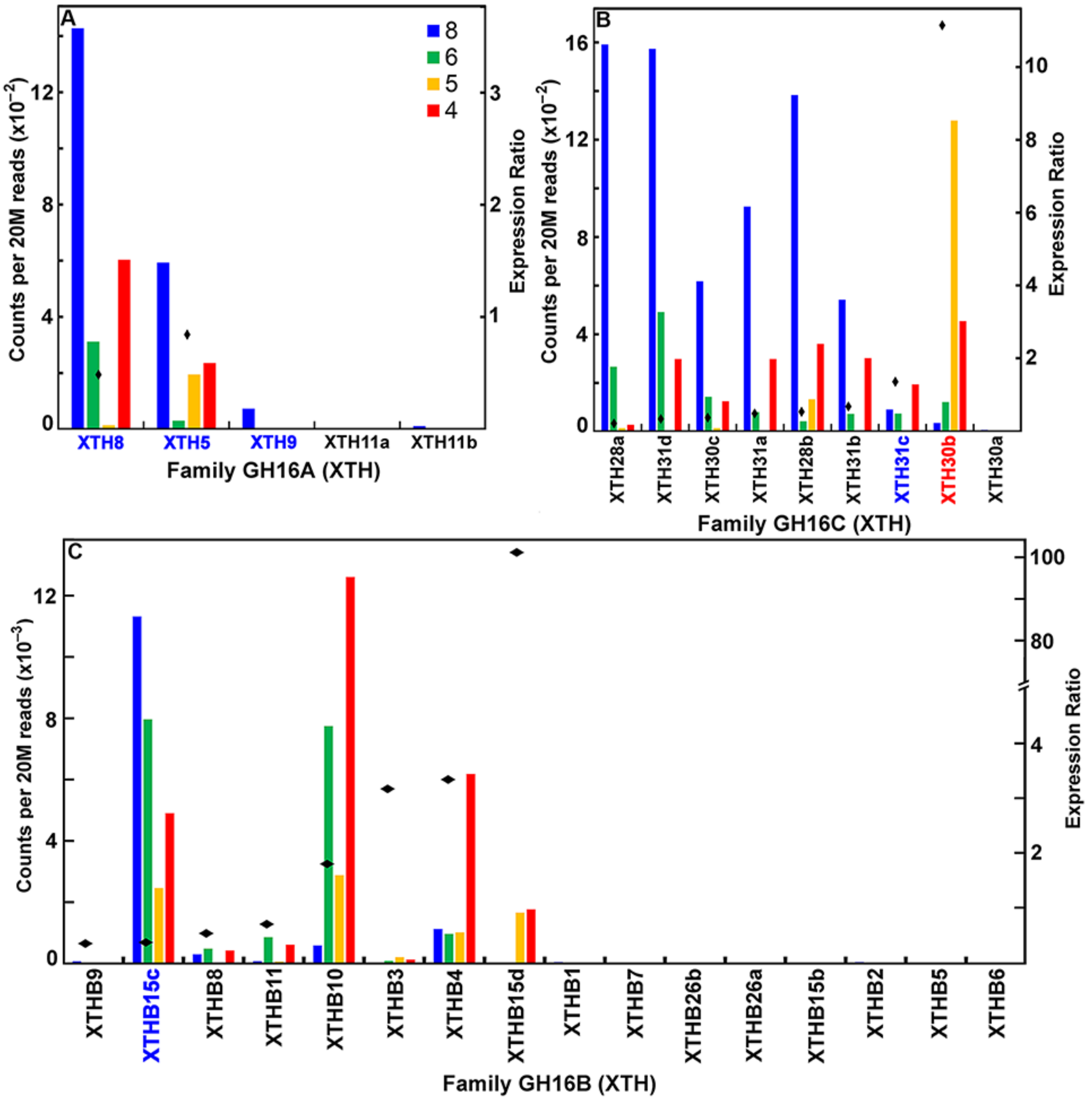

Supplemental Figure S11. Differential expression of families of the maize B73 Family GH16 xyloglucan endo-β-D-glucan transglucosylase/hydrolase superfamily. Expression ratios and putative Arabidopsis orthologs were determined as described in the legend of Figure S2. **(A)** Family GH16 Group A (XTHA). **(B)** GH16 Group C (XTHC). **(C)** GH16 Group B (XTHB).
