# Supplementary material for: Evolution of the Cell Wall Gene Families of Grasses
Source: Front Plant Sci. 2019 Oct 4;10:1205. doi: 10.3389/fpls.2019.01205 (PMC6805987; doi:10.3389/fpls.2019.01205)
Supplement: Supplementary file 2 [file DataSheet_2.pdf]

Table S1. Cell wall-related genes of maize B73 and their expression in developing stems. Expression ratio is calculated for genes with ≥500 reads per 20 M as the sum of the reads of Internodes 4 an 5 divided by the sum of reads of Internodes 6 and 8. Arabidopsis genes are color-coded to to reflect expression during floral stem development as primarily during primary wall formation (blue), secondary wall formation (red), or constitutive (purple). Putative orthologous maize genes closest in sequence to Arabidopsis and expressed during primary wall formation (blue) or secondary wall formation (red) and the Arabidopsis homologs are in bold.

Family	Maize GDBv2	Ratio of Expression	Potential Arabidopsis Orthologs	Total Expression Internodes 4-8 Reads per 20 M
**Sucrose synthases**				
**Sus6a**	GRMZM2G045171	0.06	At5g37180, **At1g73370**	586
**Sus6b**	GRMZM2G060659	0.08	At5g37180, **At1g73370**	3,799
**Sus4a**	GRMZM2G152908	0.42	**At5g20830, At3g43190**	453,510
SusL3	GRMZM2G011240	0.44	None	944
**Sus4b**	GRMZM2G089713	1.32	**At5g20830, At3g43190**	1,676
SusL1	GRMZM2G318780	1.73	None	4,572
SusL2	GRMZM2G392988	1.90	None	1,936
Sus3	GRMZM2G311182	2.77	At4g02280	3,211
**Nucleotide-sugar interconversion**				
AUD1c	GRMZM2G007404	1.16	At3g62830, **At2g47650**	4,254
AUD1b	GRMZM2G007195	2.00	At3g62830, At2g47650	26,065
AUD1a	GRMZM2G381473	2.45	At3g62830, At2g47650	1,023
AUD3c	GRMZM2G359234	3.37	At3g53520	19,639
AUD3b	GRMZM2G370048	3.73	At3g53520	14,561
AUD3a	GRMZM2G347717		At3g53520	149
AUD1d	GRMZM2G382038		None	88
SUD3b	GRMZM2G165357	0.98	At2g28760	46,682
SUD3a	GRMZM2G044027	1.16	At2g28760	65,128
**AXS1**	GRMZM2G063949	0.36	At1g08200, **At2g27860**	10,542
GAE6e	GRMZM2G052357	0.04	None	1,367
GAE6b	GRMZM2G170336	0.56	None	2,814
**GAE1b**	GRMZM2G110558	0.60	**At4g30440**	6,346
**GAE1c**	GRMZM2G042179	0.76	**At4g30440**	4,881
GAE6a	GRMZM2G017678	1.82	None	1,329
GAE6c	GRMZM2G429118	4.57	None	1,169
GAE6d	GRMZM2G161233		None	110
GAE1a	GRMZM2G455306		At4g30440	87
GAE6f	GRMZM2G163610		None	7
GER1	GRMZM2G029856		At1g17890, At1g73250	22
**GME1a**	GRMZM2G138907	1.17	**At5g28840**	4,634
GME1b	GRMZM2G124434	3.13	At5g28840	8,356
GMD1	GRMZM2G115124		At3g51160, At5g66280	351
RHM1b	GRMZM2G166767	0.99	None	11,146
RHM1c	GRMZM2G031311	1.01	None	918
RHM1a	GRMZM2G044281	7.14	None	4,703
**UER1**	GRMZM2G072911	0.56	**At1g63000**	15,571
UGD3b	GRMZM5G862540	1.26	At5g15490, At3g29360, At5g39320	99,923
UGD3a	GRMZM2G328500	2.24	At5g15490, At3g29360, At5g39320	10,479
UGD4	GRMZM2G058244		At1g26570	147
**UGE4**	GRMZM2G138410	0.13	**At1g64440**	1,424
UGE2	GRMZM2G038598	11.92	At4g23920, At4g10960	1,982
UGE3	GRMZM2G052336		At1g12780, At1g63180	129
UXE4b	GRMZM2G000632	1.13	None	2,821
UXE4c	GRMZM2G040397	1.62	None	13,300
UXE4a	GRMZM5G830983	1.63	None	3,946
UXE4d	GRMZM2G145460		None	434

RGP/UAM5c	GRMZM2G456174	0.49	None	540
**RGP/UAM3a**	GRMZM2G045287	0.71	**At3g08900**	3,023
RGP/UAM1a	GRMZM2G087326	2.53	At3g02230, At5g15650	48,636
RGP/UAM5a	GRMZM2G173341	3.04	At5g16510	41,258
RGP/UAM1b	GRMZM2G073725	3.10	At3g02230, At5g15650	78,255
RGP/UAM3b	GRMZM5G830681		At3g08900	461
RGP/UAM5d	GRMZM2G481027		None	1
RGP/UAM4	GRMZM2G034945		None	0
RGP/UAM5b	GRMZM2G046436		None	0
**Nucleotide-sugar transporters**				
Group I				
**UXT3b**	GRMZM2G116053	0.60	**At1g06890, At2g30460**	7,912
**UXTL2a**	GRMZM2G012992	0.75	**At5g55950**	1,753
**URGT3a**	GRMZM5G823629	0.88	**At4g39390**	3,348
**URGT3b**	GRMZM2G089630	0.88	**At4g39390**	3,684
**UXT3a**	GRMZM2G081848	0.94	**At1g06890, At2g30460**	4,375
UXT1a	GRMZM2G068714	1.01	None	4,905
URGTL1	GRMZM2G147446	1.09	None	3,827
URGTL2	GRMZM2G071378	1.11	None	5,523
**UXT2a**	GRMZM2G063253	1.16	**At1g06890, At2g30460**	19,870
**UXT2b**	GRMZM2G063511	1.30	**At1g06890, At2g30460**	17,915
**URGT3b'**	AC194970.5_FG005	1.49	**At5g42420**	8,053
UXT1c	GRMZM5G828581	2.52	None	12,570
URGT3a'	GRMZM2G055216	2.55	At5g42420	13,691
UXT1d	GRMZM2G048434	2.64	None	7,472
UXT1b	GRMZM2G122618	3.40	None	20,006
URGTL3	GRMZM2G021971		None	472
URGTL4	GRMZM2G014610		None	390
UXTL2b	GRMZM2G075372		At5g55950	313
Group II				
**UTR1a**	GRMZM2G045686	0.64	**At2g02810, At1g14360**	630
**UTR1b**	GRMZM2G176029	0.82	**At2g02810, At1g14360**	5,288
**UTR5a**	GRMZM2G005163	0.84	**At3g46180, At5g59740**	818
UTR5b	GRMZM2G180519		At3g46180, At5g59740	288
UTR2	GRMZM2G143187		At1g12600, At4g23010	169
Group III				
NSTL4b	AC214266.3_FG002	0.14	At4g35335	952
**GONST4a**	GRMZM2G088849	0.46	**At5g19980**	912
UTR6c	GRMZM5G842886	0.73	None	2,050
**GONST3a**	GRMZM2G173067	0.77	**At1g76340**	2,825
**GONST4b**	GRMZM2G058120	0.98	**At5g19980**	1,489
**UTR6b**	GRMZM2G001033	1.20	**At2g43240, At3g59360**	8,715
**GONST3b**	GRMZM2G013349	1.33	**At1g76340**	7,825
**ROCK1a**	GRMZM2G025236	1.49	**At5g65000**	3,758
**ROCK1b**	GRMZM2G403915	1.54	**At5g65000**	2,348
**UTR6a**	GRMZM2G172647	1.66	**At2g43240, At3g59360**	8,231
UGNT1a	GRMZM2G133226	1.98	At4g32272	4,275
NSTL3	GRMZM2G093598	2.04	At5g41760	4,103
UTR7a	GRMZM2G135476	2.30	At4g31600	1,281
UGNT1b	GRMZM2G020976	2.71	At4g32272	5,206
GONST1	GRMZM5G863229	2.77	At2g13650	1,523
NSTL4a	GRMZM2G007909		At4g35335	62
NSTL4c	GRMZM2G421565		None	52
UTR7c	GRMZM2G050157		At4g31600	29
UTR7b	GRMZM2G375847		At4g31600	28
GONST2	GRMZM2G081105		At1g07290	27
Group IV				
**TPTa**	GRMZM2G083344	0.42	**At5g46110**	7,910
PPT1b	GRMZM2G103047	0.69	At5g33320	9,804
PPT1c	GRMZM2G047404	0.79	At5g33320	14,665
GPT1b	GRMZM2G180720	1.34	None	20,036
GPT1a	GRMZM2G431314	1.40	None	11,170
PPT1a	GRMZM2G174107	1.62	At5g33320	17,856
GPT1c	GRMZM2G009223	2.01	None	4,589
TPTb	GRMZM2G070605	3.36	At5g46110	11,184
TPTc	GRMZM2G059412	3.70	None	7,851
PPT2	GRMZM2G066413		At3g01550	95
Group V				
**GalT1a**	GRMZM2G127591	0.25	**At1g77610, At1g21870**	1,441
UUAT1b	GRMZM2G443265	2.16	At5g05820, At3g11320	8,853

UUAT1a	GRMZM2G447617	2.81	At5g05820, At3g11320	9,936
**GalT1b**	GRMZM2G040033	1.12	**At1g77610, At1g21870**	355
NSTL10	AC219006.2_FG006		None	51
Group VI				
**UAfT1**	GRMZM2G002699	0.28	**At1g48230, At3g17430**	2,705
UAfT2a	GRMZM2G090300	0.98	None	12,702
UAfT2b	GRMZM2G062555	1.12	None	8,891
**NSTL17**	GRMZM2G022793	1.27	**At1g53660, At3g14410**	3,089
UAfT2c	GRMZM2G130558	7.37	None	11,044
UAfT3	GRMZM2G178734		At1g06470	479
UAfT2d	AC204359.3_FG005		None	0
**Cellulose synthases**				
**CesA8a**	GRMZM2G082580	0.68	**At4g39350, At2g21770, At5g09870, At5g64740**	15,889
**CesA8b**	GRMZM2G177631	0.75	**At4g39350, At2g21770, At5g09870, At5g64740**	35,371
**CesA4**	GRMZM2G424832	0.83	**At4g32410, At2g25540**	16,307
**CesA3**	GRMZM2G039454	0.85	**At4g32410, At2g25540**	9,360
**CesA5**	GRMZM2G111642	0.91	**At4g32410, At2g25540**	6,272
**CesA7b**	GRMZM2G028353	1.00	**At4g39350, At2g21770, At5g09870, At5g64740**	25,358
**CesA7a**	GRMZM2G025231	1.17	**At4g39350, At2g21770, At5g09870, At5g64740**	44,559
**CesA9**	GRMZM2G018241	1.25	**At4g32410, At2g25540**	25,287
**CesA6**	GRMZM2G113137	1.56	**At4g39350, At2g21770, At5g09870, At5g64740**	29,711
**CesA1**	GRMZM2G112336	1.78	**At4g32410, At2g25540**	48,105
**CesA2**	GRMZM2G027723	1.85	**At4g32410, At2g25540**	59,420
CesAL4	GRMZM2G150404	1.97	At4g32410, At2g25540	9,513
**CesA10**	GRMZM2G445905	3.35	**At5g44030**	24,680
**CesA12b**	GRMZM2G002523	4.38	**At5g17420**	7,981
**CesA11b**	GRMZM2G055795	4.62	**At4g18780**	55,576
**CesA11a**	GRMZM2G037413	4.62	**At4g18780**	55,656
**CesA12a**	GRMZM2G142898	5.94	**At5g17420**	10,761
CesAL1	GRMZM2G104092		At4g32410, At2g25540	479
CesAL3	GRMZM2G378836		At4g32410, At2g25540	113
CesAL2	GRMZM2G349834		At4g18780	10
**Cellulose-synthase-like genes**				
Cellulose synthase-likeA (CslA)				
CslAL1	GRMZM2G010142	0.16	None	1,447
**CslA2**	GRMZM2G105631	0.24	**At5g22740**	9,071
CslAL2	GRMZM2G115772	0.31	None	1,566
CslAL5	GRMZM2G020742	0.37	None	752
CslAL4	GRMZM2G405567	0.42	None	931
CslAL7	GRMZM2G099088	0.45	None	3,021
CslAL3	GRMZM2G178880	0.49	None	726
CslAL6	GRMZM2G108600	0.56	None	2,808
CslA9	GRMZM2G443715	1.08	At5g03760	1,118
CslAL8	GRMZM2G107754		None	255
Cellulose synthase-likeC (CslC)				
CslC12e	GRMZM2G454081	0.37	None	2,471
CslC12d	GRMZM2G027794	0.47	None	2,382
**CslC5a**	GRMZM2G173759	0.53	**At4g31590, At2g24630**	5,931
CslC12a	GRMZM2G028286	0.71	At4g07960	6,244
CslC12b	AC183932.3_FG007	0.81	None	5,097
CslC12c	GRMZM2G074792	0.83	None	12,428
CslC5b	GRMZM2G142685		At4g31590, At2g24630	55
CslC5c	GRMZM2G135286		At4g31590, At2g24630	9
Cellulose synthase-likeD (CslD)				
**CslD5**	GRMZM2G015886	0.12	**At1g02730**	2,674
CslD3a	GRMZM5G870176	2.25	At3g03050	26,251
CslD4	GRMZM2G044269		At4g38190	187
CslD1	GRMZM2G061764		At3g03050	166
CslD3b	GRMZM2G436299		At2g33100	156
Cellulose synthase-likeE (CslE)				
**CslE1a**	GRMZM2G014558	0.01	**At1g55850**	516
**CslE1b**	GRMZM2G012044	1.83	**At1g55850**	1,520
Cellulose synthase-likeF (CslF)				
CslF3	GRMZM2G113432	0.05	None	1,024
CslF7	GRMZM2G339645	0.72	None	996
CslF2	GRMZM2G110145	0.90	None	20,841
CslF4	GRMZM2G122277	0.98	None	21,783
CslF5	GRMZM2G150404	1.97	None	9,513
CslF8	GRMZM2G367267		None	181

CslF9	GRMZM2G378836		None	113
CslF1	GRMZM2G103972		None	37
CslF6	GRMZM2G164761		None	7
Cellulose synthase-likeG (CslG)				
CslG1	GRMZM2G122431		At4g23990	55
**Callose synthases**				
**GSL1**	GRMZM2G022856	0.13	**At4g04970**	3,323
**GSL12b**	GRMZM2G430680	0.13	**At5g13000**, At5g36870, At2g31960, At1g05570	7,585
GSL12d	GRMZM2G111529	0.17	None	1,563
**GSL7a**	GRMZM2G084802	0.50	**At1g06490, At3g59100**	2,270
**GSL7b**	GRMZM2G465764	0.60	**At1g06490, At3g59100**	4,337
**GSL8**	GRMZM2G326643	0.75	**At2g36850**	15,807
**GSL12a**	GRMZM5G840560	1.15	**At5g13000, At5g36870, At2g31960**, At1g05570	7,111
GSL12f	GRMZM2G453794	1.37	None	12,463
GSL12c	GRMZM2G180951	4.81	None	5,832
GSL12e	GRMZM2G341918	5.04	None	702
GSL2	GRMZM2G353905		At2g13680	285
GSL12g	GRMZM2G023003		None	187
**Glycosyl Transferase Family 8 (GT8)**				
Group A				
**GUX1b**	GRMZM2G002023	0.23	At3g18660, **At1g77130**	5,810
**GUX1a**	GRMZM2G058472	0.64	At3g18660, **At1g77130**	1,142
GUX1c	GRMZM2G365544	1.11	At3g18660, At1g77130	7,863
GUX1d	GRMZM2G135743	1.29	At3g18660, At1g77130	14,060
GUX2	GRMZM2G109431		At4g33330	429
GUX1e	GRMZM2G031581		None	111
GUX1f	GRMZM2G441987		None	15
Group C				
GATL7c	GRMZM2G000587	0.11	None	1,200
**GATL3a**	GRMZM2G057930	0.23	**At1g13250**	513
GATL7d	GRMZM2G142709	0.80	None	4,318
**GATL2**	GRMZM2G149024	1.14	**At3g50760**, At1g19300	4,125
**GATL3b**	GRMZM2G468661	1.21	**At1g13250**	563
GATL7b	GRMZM2G300692	3.25	At3g62660, At4g02130, At1g02720	2,325
GATL4a	GRMZM2G108576		At3g06260	327
GATL4b	GRMZM2G019583		At3g06260	81
GATL7a	GRMZM2G443785		At3g62660, At4g02130, At1g02720	3
GATL7e	GRMZM2G469828		None	0
Group D				
**GAUT7a**	GRMZM2G036918	0.22	**At2g38650**	4,931
GAUT9a	AC209664.3_FG002	0.30	None	1,935
**GAUT1d**	GRMZM2G076276	0.31	**At3g61130**	3,610
**GAUT1a**	GRMZM2G386971	0.37	**At3g61130**	5,846
**GAUT7c**	GRMZM2G045467	0.38	**At2g38650**	2,095
**GAUT1c**	GRMZM2G147145	0.42	**At3g61130**	5,517
**GAUT15**	GRMZM2G014770	0.46	**At3g58790**	1,313
**GAUT7b**	GRMZM2G008501	0.56	**At2g38650**	2,798
**GAUT13b**	GRMZM2G107854	0.58	**At3g01040, At5g15470**	2,518
**GAUT1b**	GRMZM2G379577	0.58	**At3g61130**	3,230
**GAUT6**	GRMZM2G135195	0.60	At2g30575, **At1g06780**	1,648
**GAUT4b**	GRMZM2G048008	0.60	**At5g47780**	1,644
**GAUT13c**	GRMZM2G113506	0.76	**At3g01040, At5g15470**	5,321
**GAUT8**	GRMZM2G130046	0.81	**At3g25140**	8,229
**GAUT10b**	GRMZM2G143102	0.86	**At2g20810**	2,732
**GAUT3**	GRMZM2G028501	0.97	**At4g38270**	1,383
**GAUT13a**	GRMZM2G098434	1.06	**At3g01040, At5g15470**	6,526
**GAUT4a**	GRMZM2G391000	1.39	**At5g47780**	7,202
**GAUT10a**	GRMZM2G063519	1.46	**At2g20810**	2,381
GAUT11b	AC177908.3_FG002	2.62	At1g18580	4,342
GAUT11c	GRMZM2G078890	3.01	**At1g18580**	2,440
GAUT11a	GRMZM2G151015		At1g18580	324
GAUT9b	GRMZM2G166940		At3g25140, At3g02350	171
Group E				
**IPUT1**	GRMZM2G166903	1.79	**At5g18480**	6,896
PGSIP7a	GRMZM2G091581	2.42	At2g35710, At4g16600	6,688
PGSIP7b	GRMZM2G462261	1.85	At2g35710, At4g16600	7,729
PGSIP7c	GRMZM2G114772	13.05	At2g35710, **At4g16600**	2,004
PGSIP7d	GRMZM2G026889	10.15	At2g35710, At4g16600	989

**Glycosyl Transferase Family 31 (GT31)**				
Group A				
**GT31A1**	GRMZM2G059013	0.37	**At1g33250**	1,157
GT31A8	GRMZM2G426067	0.42	None	571
GT31A9	GRMZM2G107501	0.47	None	527
GT31A10	GRMZM2G061202	1.39	At3g11420, At2g37730	3,565
GT31A3	GRMZM2G017533	3.60	At4g15240, At1g05280	1,991
GT31A6	GRMZM2G102657		None	383
GT31A7	GRMZM2G098676		None	345
GT31A2	GRMZM2G103785		At4g15240, At1g05280	337
GT31A4	GRMZM2G040068		At5g41460, At4g23490, At4g11350	15
GT31A5	GRMZM2G071325		At5g41460, At4g23490, At4g11350	11
Group B				
**GALT2b**	GRMZM2G131329	0.32	**At4g21060**	1,406
GALT4f	GRMZM2G149935	0.39	None	3,305
**GALT3**	GRMZM2G160958	0.57	**At3g06440**	2,172
**GALT2a**	GRMZM2G063688	0.58	**At4g21060**	2,648
GALT1a	GRMZM2G042607	0.81	At1g26810	2,871
GALT1b	GRMZM2G164072	0.95	At1g26810	3,166
GALT4e	GRMZM2G176630	1.81	None	3,341
**GALT4c**	GRMZM2G054350	1.90	**At1g27120, At1g74800, At5g62620**	902
GALT4d	GRMZM2G176774		At1g27120, At1g74800, At5g62620	452
GALT4a	GRMZM2G149841		**At1g27120, At1g74800, At5g62620**	245
GALT1c	AC206332.3_FG005		None	44
GALT4b	GRMZM2G025048		At1g27120, At1g74800, At5g62620	17
GroupC				
**GT31C2**	GRMZM2G130449	0.71	**At1g53290, At3g14960**	2,761
GT31C1	GRMZM2G022768	0.90	At2g26100	3,744
**GT31C3**	GRMZM5G838414	1.26	**At1g53290, At3g14960**	1,969
GroupD				
**HPGT2**	GRMZM2G000393	0.23	**At4g32120, At2g25300**	2,837
**HPGT1a**	GRMZM2G116971	0.67	**At5g53340**	2,953
**HPGT1b**	GRMZM2G153760	0.87	**At5g53340**	3,793
GroupE				
**GALT31A**	GRMZM2G144873	0.31	**At1g32930**	2,931
**GT31E3**	GRMZM2G007185	0.38	**At1g11730**	649
GT31E2	GRMZM2G132866	0.94	At1g11730	7,537
**GT31E6**	GRMZM2G070708	0.97	**At1g77810, At1g22015, At1g33430**	2,727
**GT31E5**	GRMZM2G013079	1.10	**At1g77810, At1g22015, At1g33430**	2,567
GT31E1	GRMZM2G080231	1.49	At4g26940, At2g32430, At1g05170	894
GT31E4	GRMZM2G133613		At1g77810, At1g22015, At1g33430	341
GroupF				
GT31F4	GRMZM2G072406	10.12	None	1,303
GT31F5	GRMZM2G057779	58.15	None	1,691
GT31F1	GRMZM2G037998		None	116
GT31F2	GRMZM2G181358		None	5
GT31F3	GRMZM2G011626		None	2
**Glycosyl Transferase Family34 (GT34)**				
**XXT2b**	GRMZM2G129302	0.19	**At3g62720, At4g02500**	708
**XXTL2**	GRMZM2G154124	0.36	**At4g37960**	2,215
**XXT3a**	GRMZM5G876303	0.76	**At5g07720, At1g18690, At1g74380**	637
XXT2c	GRMZM2G115762		At3g62720, At4g02500	373
XXTL1b	GRMZM2G123355		At2g22900	353
XXT2a	GRMZM2G031453		At3g62720, At4g02500	137
XXTL1c	GRMZM2G435120		At2g22900	55
GT34-3	GRMZM2G072589		None	20
XXT3f	GRMZM2G159208		None	11
XXT3e	GRMZM2G044394		None	10
XXT3d	GRMZM2G445201		None	8
XXT3c	GRMZM2G159193		At5g07720, At1g18690, At1g74380	6
GT34-2	GRMZM2G383790		None	4
XXT3b	GRMZM2G044377		At5g07720, At1g18690, At1g74380	4
XXTL1d	GRMZM2G423930		At2g22900	3
GT34-1	GRMZM2G164283		None	1
GT34-4	GRMZM2G434155		None	0
XXTL1a	AC186147.3_FG008		At2g22900	0
**Glycosyl Transferase Family 37 (GT37)**				
**FUT2**	GRMZM2G404762	0.21	**At2g03210**	758
FUTL11	GRMZM2G015983	7.13	None	1,642

FUT3a	GRMZM2G103128		At1g74420	261
FUTL2	GRMZM5G870571		None	133
FUT3b	GRMZM2G118312		At1g74420	75
FUTL4	GRMZM5G858456		None	58
FUTL8	GRMZM2G104032		None	38
FUTL3	GRMZM2G127184		None	36
FUTL9	GRMZM2G387087		None	26
FUTL7	GRMZM2G470010		None	11
FUTL1	GRMZM2G436950		None	9
FUTL12	GRMZM2G080696		None	9
FUTL5	GRMZM2G015654		None	7
FUTL6	GRMZM2G333274		None	4
FUTL10	GRMZM2G096268		None	0
FUT3c	GRMZM5G857459		None	0
FUT3d	GRMZM2G471594		None	0
**Glycosyl Transferase Family43 (GT43)**				
IRX9Le	GRMZM2G150264	1.10	None	2,008
IRX14d	GRMZM2G160585	1.24	At5g67230, At4g36890	15,002
IRX9Lb	GRMZM2G068350	1.27	At1g27600	1,362
IRX9c	GRMZM2G012874	1.63	At2g37090	6,589
IRX9Ld	GRMZM2G103628	1.73	None	6,078
IRX9Lc	GRMZM2G037469	1.90	None	3,981
IRX9La	GRMZM2G179504	2.06	At1g27600	531
**IRX14b**	GRMZM2G150302	2.27	At5g67230, **At4g36890**	7,031
IRX14c	GRMZM5G869532	2.50	**At5g67230**, At4g36890	1,126
**IRX9a**	GRMZM2G118959	2.92	**At2g37090**	2,901
**IRX9b**	GRMZM2G001079	3.01	**At2g37090**	7,025
**IRX14a**	GRMZM2G113655	3.19	At5g67230, **At4g36890**	15,022
IRX9d	GRMZM2G076307		None	336
IRX9Lg	GRMZM2G356579		None	153
IRX9Lh	GRMZM2G025302		None	22
IRX9Lf	GRMZM2G448938		None	1
**Glycosyl Transferase Family47 (GT47)**				
GroupA				
**MUR3a**	GRMZM2G137779	0.67	**At2g20370**	626
**MUR3b**	GRMZM2G430995	0.72	**At2g20370**	1,340
**MUR3c**	GRMZM2G332548	0.97	**At2g20370**	2,979
**GT47A14**	GRMZM2G089248	1.68	**At4g22580**	670
GT47A16	GRMZM2G021401		At4g22580	137
GT47A13	GRMZM2G161540		At4g22580	47
GT47A11	GRMZM2G317731		None	29
GT47A18a	GRMZM2G303135		At5g62220	26
GT47A4	GRMZM5G865819		None	20
GT47A10	GRMZM2G464680		None	16
GT47A9	GRMZM2G075436		None	9
GT47A18b	GRMZM2G305146		At5g62220	7
MUR3d	GRMZM2G075492		At2g20370	5
MUR3e	GRMZM2G016338		At2g20370	5
GT47A7	GRMZM2G470309		None	0
GT47A8	GRMZM2G406662		None	0
GT47A15	GRMZM2G175417		At4g22580	0
GT47A1	GRMZM2G172602		None	0
GT47A2	GRMZM5G827810		None	0
GT47A3	GRMZM2G142214		None	0
GT47A5	GRMZM2G315692		None	0
GT47A6	GRMZM2G435276		None	0
GT47A12	AC177899.2_FG009		None	0
Group B				
EDA5d	GRMZM2G022398	0.29	At5g03795	958
EDA5c	GRMZM2G009690	0.46	At3g03650, At3g45400	502
GT47B3	GRMZM2G139691	0.55	At5g16890	1,505
**GT47B4**	GRMZM2G149932	0.69	**At1g34270**	1,284
EDA5a	GRMZM2G054703	0.71	At3g03650, At3g45400	1,112
EDA5b	GRMZM2G127416	0.87	At3g03650, At3g45400	1,548
**ARAD1**	GRMZM2G060579	1.14	**At2g35100**, **At5g44930**	2,926
**GT47B2**	GRMZM2G158496	1.40	**At1g67410**	2,883
GT47B1	GRMZM2G139355		At1g74680	3
Group C				
GT47C1	GRMZM2G149189		At5g20260	50

GT47C3	GRMZM2G325561		At5g25310	14
GT47C2	GRMZM2G001938		None	9
Group D				
**GT47D3**	GRMZM2G018962	0.12	**At4g38040**	933
**GT47D4**	GRMZM2G000976	0.26	**At4g38040**	966
**GT47D2**	GRMZM2G322047	0.55	**At5g19670**	758
**GT47D5**	GRMZM2G076688	0.70	**At1g21480**	2,075
GT47D1	GRMZM2G048010		At4g16745	8
Group E				
IRX10-1f	GRMZM2G056702	0.35	None	6,133
IRX10-1g	GRMZM2G023020	0.45	None	2,977
IRX10-1i	GRMZM2G134308	0.60	None	990
IRX10-1h	GRMZM2G448834	0.65	None	1,542
FRA8a	GRMZM2G113245	1.06	At2g28110, At5g22940	597
**IRX10-1c**	GRMZM2G000581	2.73	**At1g27440**, At5g61840	5,615
IRX10-1e	GRMZM2G059845	3.07	None	7,430
**IRX10-1a**	GRMZM2G059825	3.95	**At1g27440**, At5g61840	32,830
**IRX10-1d**	GRMZM5G898668	4.90	**At1g27440**, At5g61840	10,535
FRA8b	GRMZM2G035377		At2g28110, At5g22940	153
IRX10-1b	GRMZM2G152029		At1g27440, At5g61840	53
**Glycosyl Transferase Family 61 (GT61)**				
**MUCI21d**	GRMZM2G139574	0.14	**At2g03370, At2g03360**	1,724
**MUCI21e**	GRMZM5G869788	0.26	**At2g03370, At2g03360**	785
XYLTa	GRMZM2G096946	0.28	None	4,530
GT61-8	GRMZM2G176576	0.41	None	6,246
GT61-13	GRMZM2G024444	0.54	None	627
**GT61-6**	GRMZM2G130800	0.72	**At3g18180**, At3g18170	3,333
**XYLTh**	GRMZM2G419267	0.93	**At5g55500**	2,405
GT61-18	GRMZM2G476699	1.09	None	1,365
GT61-11	GRMZM2G062552	1.57	None	2,479
**GT61-5**	GRMZM5G875864	1.63	**At3g18180**, At3g18170	2,469
**MUCI21a**	GRMZM2G131055	2.15	**At3g10320**, At3g57380, **At2g41640**	3,120
GT61-9	GRMZM2G098793	3.33	None	12,034
GT61-20	GRMZM2G074896	3.39	None	3,761
**MUCI21c**	GRMZM2G459363	4.42	**At3g10320**, At3g57380, **At2g41640**	1,280
GT61-19	GRMZM2G354610	8.34	None	1,716
GT61-15	GRMZM2G090584	9.00	None	5,180
GT61-14	GRMZM2G412986		None	397
GT61-12	GRMZM2G094579		None	295
XYLTc	GRMZM2G410916		None	253
GT61-3	GRMZM2G022442		At3g18180, At3g18170	239
GT61-7	GRMZM2G447347		None	236
XYLTd	GRMZM2G397391		None	96
GT61-16	GRMZM2G336865		None	51
GT61-1	GRMZM2G105221		At2g03370, At2g03360	43
GT61-4	GRMZM2G700386		At3g18180, At3g18170	40
GT61-10	GRMZM2G099082		None	32
XYLTg	GRMZM2G008316		None	29
MUCI21b	GRMZM2G136580		At3g10320, At3g57380, At2g41640	22
XYLTb	GRMZM2G104829		None	3
XYLTf	GRMZM2G464895		None	3
XYLTe	GRMZM2G065939		None	2
GT61-17	GRMZM2G336929		None	1
GT61-2	GRMZM2G451975		At2g03370, At2g03360	0
**Glycosyl Transferase Family 77 (GT77)**				
**RRA1a**	GRMZM2G053946	0.20	At1g75120, **At1g75110**, **At1g19360**	3,170
GT77-18	GRMZM2G421126	0.45	None	726
**RGXT3**	GRMZM2G085042	0.50	At4g01770, **At4g01750, At4g01220**, At1g56550	1,479
GT77-2	GRMZM2G014187	0.76	At2g02061, At1g14590	968
**XEG113**	GRMZM2G011912	1.74	**At2g35610**	2,908
RRA1b	GRMZM2G028905	2.06	At1g75120, At1g75110, At1g19360	2,174
RRA1c	GRMZM2G172726		At1g75120, At1g75110, At1g19360	335
GT77-8	GRMZM2G104231		None	293
GT77-9	GRMZM2G421579		None	236
GT77-11	GRMZM2G313481		None	176
GT77-3	GRMZM2G008064		At2g02061, At1g14590	144
GT77-10	GRMZM2G146158		None	135
GT77-1	GRMZM2G378073		At2g02061, At1g14590	75
GT77-12	GRMZM2G129114		None	73

GT77-13	GRMZM2G180324		None	68
GT77-4	GRMZM2G011383		At1g28710, At1g28700, At1g28695	23
GT77-16	GRMZM2G180330		None	13
GT77-7	GRMZM2G144034		None	13
GT77-17	GRMZM2G001497		None	8
GT77-15	GRMZM2G481052		None	2
GT77-5	GRMZM2G150215		None	2
GT77-6	GRMZM2G077106		None	1
GT77-14	GRMZM2G304956		None	1
**Glycosyl Transferase Family 106 (GT106)**				
**Group A**				
RRT-L1a	GRMZM2G087513	0.55	At5g64600	796
RRT1a	GRMZM2G104511	1.33	At5g15740, At3g02250	6,284
RRT1b	GRMZM2G089854	3.59	At5g15740, At3g02250	1,103
RRT-L2	GRMZM6G438921		At1g22460	0
**Group B**				
**MSR1a**	GRMZM2G080565	0.50	**At1g52630**	1,163
MSR3a	GRMZM2G025671	0.54	At3g03810	2,421
MSR4b	GRMZM2G050649	0.60	At1g04910	3,775
MSR4a	GRMZM2G320802	0.66	At1g04910	3,676
MSR3b	GRMZM2G025867	0.84	At3g03810	4,357
MSR3c	GRMZM2G014793	0.88	At3g03810	4,731
**Group C**				
**PAGR1b**	GRMZM2G134156	0.36	**At3g26370**	824
**PAGR-L3**	GRMZM2G054028	0.44	**At1g11990, At1g62330**	580
**PAGR1a**	GRMZM2G139657	0.76	**At3g26370**	1,698
PAGR-L1	GRMZM2G057037	1.64	At4g24530, At5g65470	3,494
PAGR-L5	GRMZM2G124872		At1g29200	355
PAGR-L9a	GRMZM2G419739		At2g01480, At1g14970	157
PAGR1c	GRMZM5G817948		At3g26370	98
PAGR-L8	ACF80826		At1g35510	0
PAGR-L9b	ACR35648		At2g01480, At1g14970	0
**Group D**				
**GT106-L1b**	GRMZM2G131699	0.50	**At1g76270, At1g20550**	2,318
GT106-L4	GRMZM2G101181	0.54	At4g16650	1,161
GT106-L1a	GRMZM2G142664	1.26	At1g76270, At1g20550	1,238
**TBL genes**				
**Group A**				
TBL6	AC233955.1_FG009		At3g62390	83
TBL5	GRMZM2G414826		At5g20590	12
**Group B**				
TBL10b	GRMZM2G004445	0.17	At3g06080, At5g19160	660
TBL7	GRMZM2G129065	0.43	At1g48880	1,107
TBLa	GRMZM2G083555	0.92	At3g06080, At5g19160	901
TBL10a	GRMZM2G025250		At3g06080, At5g19160	434
TBL9	GRMZM2G321394		At5g06230, At3g11570	4
**Group C**				
TBL16a	GRMZM2G094500	0.16	At5g20680	614
TBL13b	GRMZM2G010714	0.19	At2g14530	661
TBL14	GRMZM2G043240	1.17	At5g64020, At2g37720	4,115
TBL12a	GRMZM2G117804		At5g64470	444
TBL13a	GRMZM2G395061		At2g14530	141
TBL12b	GRMZM2G098438		At5g64470	99
TBL16b	GRMZM2G318337		At5g20680	0
TBL16c	GRMZM2G094482		At5g20680	0
**Group D**				
TBL20c	GRMZM2G004183	0.41	At3g02440, At5g15890, At5g15900	6,379
TBL25b	GRMZM2G061283	0.62	At1g01430, At4g01080, At4g11090, At4g23790	1,446
TBL25a	GRMZM2G131152	0.67	At1g01430, At4g01080, At4g11090, At4g23790	2,074
TLS7	GRMZM2G096169	0.69	At5g51640, At4g25360	2,312
AXY4a	GRMZM2G122073	4.25	At1g70230	2,459
AXY4c	GRMZM2G039525		At1g70230	234
AXY4b	GRMZM2G340933		At1g70230	138
TBL20b	GRMZM2G305439		At3g02440, At5g15890, At5g15900	132
TBL20d	AC216886.3_FG002		At3g02440, At5g15890, At5g15900	108
TBLc	GRMZM2G047941		None	90
TBLb	GRMZM2G138771		None	54
AXY4L	GRMZM2G107373		At3g28150	32
TBL20a	GRMZM2G164715		At3g02440, At5g15890, At5g15900	30

TBLd	GRMZM2G005454		None	0
TBLe	AC216886.3_FG001		None	0
**Group E**				
TBLf	GRMZM2G117742	0.26	None	2,571
TBLs	GRMZM2G038855	0.31	None	693
TBL1a	GRMZM2G033805	0.32	At3g12060, At5g06700	5,973
TBLt	GRMZM5G898867	0.44	None	2,459
TBLj	GRMZM2G047298	0.64	None	3,368
TBL28b	AC208201.3_FG003	0.65	At2g40150, At3g55990	767
TBL3	GRMZM2G078469	0.69	At5g01360	551
TBL28a	GRMZM2G134341	0.73	At2g40150, At3g55990	3,191
TBLh	GRMZM2G400938	0.84	None	544
TBL1b	GRMZM2G165961	1.18	At3g12060, At5g06700	741
TBLu	GRMZM2G012021	1.22	None	3,280
TBL32b	GRMZM5G826911	1.26	At3g11030, At2g40320	7,314
TBLp	GRMZM2G411159	1.35	None	1,464
TBLq	GRMZM2G451325	1.62	None	1,430
TBLk	GRMZM2G147266	1.65	None	8,145
TBL32a	GRMZM2G139250	3.83	At3g11030, At2g40320	51,656
TBLr	GRMZM2G066165		None	456
TBL1c	GRMZM5G843892		At3g12060, At5g06700	424
TBLn	GRMZM2G040833		None	267
TBL34	GRMZM2G406974		At2g38320	242
TBLm	GRMZM2G090728		None	167
TBLo	GRMZM2G043095		None	128
TBL2	GRMZM2G077437		At1g60790	40
TBLi	GRMZM2G404081		None	38
TBLg	GRMZM2G409309		None	24
TBL45	GRMZM2G059565		At2g30010	0
**AXY Genes**				
AXY9b	GRMZM2G095698	1.63	At3g03210	2,076
**AXY9a**	GRMZM2G131221	3.29	**At3g03210**	2,884
**RWA Genes**				
RWA-L6	GRMZM2G413162	0.80	None	9,377
RWA2	GRMZM2G020721	1.74	At3g06550	15,562
RWA-L5	GRMZM2G458538	2.24	None	22,888
RWA-L1	GRMZM2G076394		None	402
RWA-L2	GRMZM2G147351		None	129
RWA-L4	GRMZM2G463445		None	79
RWA-L3	GRMZM2G370741		None	38
**BAHD Genes**				
BAHD5a	GRMZM2G050270	0.67	None	4,148
BAHD5b	GRMZM2G094428	1.37	None	6,047
BAHD3	GRMZM2G050072	1.49	None	2,631
BAHD4a	GRMZM2G060210	1.72	None	1,413
BAHD4b	GRMZM2G108714	1.77	None	3,316
BAHD8	GRMZM2G159641	5.55	None	3,918
BAHD2a	GRMZM2G375159	6.12	None	13,193
BAHD9 (ZmPM1	GRMZM2G028104	13.42	None	35,743
BAHD7	GRMZM2G130728		None	437
BAHD10	GRMZM2G107027		None	422
BAHD2b	GRMZM2G305900		None	168
BAHD1	GRMZM2G314898		None	53
**ER/Golgi-resident proteins**				
Glycosyl Transferase Family 10 (GT10)				
**FUT13**	GRMZM2G068710	0.60	**At1g71990**	4,289
FUT1b	GRMZM2G386463	0.75	None	1,699
FUT1a	GRMZM2G103939	0.81	None	3,611
Glycosyl Transferase Family 13 (GT13)				
**CGL1**	GRMZM2G426275	0.80	**At4g38240**	2,851
Glycosyl Transferase Family 14 (GT14)				
**UNE7**	GRMZM2G038898	0.30	**At3g03690**	1,324
**GT14-3**	GRMZM2G060061	0.48	**At1g71070**	1,770
**GT14-1**	GRMZM2G018869	0.65	**At1g71070**	1,753
**GLCAT14Ab**	GRMZM2G423476	0.78	**At5g39990, At5g15050**	1,145

GT14-5	GRMZM2G178571	1.07	At3g24040	6,053
GLCAT14Ad	GRMZM2G043310	1.69	None	6,993
GLCAT14Ac	GRMZM2G150323	1.83	None	7,574
GT14-2	GRMZM2G055313		At4g27480, At3g15350, At1g53100	470
GLCAT14Aa	GRMZM2G063133		At5g39990, At5g15050	49
GT14-4	GRMZM2G111428		None	3
Glycosyl Transferase Family 16 (GT16)				
**GT16**	GRMZM2G084515	0.45	**At2g05320**	604
Glycosyl Transferase Family 17 (GT17)				
GT17-2	GRMZM2G062488	0.72	None	1,429
GT17-4	GRMZM2G104118	0.97	None	6,335
GT17-3	GRMZM2G079682	1.64	None	3,307
GT17-1	GRMZM2G045435		At2g13290	337
Glycosyl Transferase Family 21 (GT21)				
**GCS**	GRMZM2G061419	0.34	**At2g19880**	4,471
Glycosyl Transferase Family 22 (GT22)				
APTG1d	GRMZM2G164175	0.38	None	1,025
**EBS3**	GRMZM2G051262	0.43	**At1g16900**	4,222
APTG1a	GRMZM2G000937	0.46	None	932
ALG12	GRMZM2G152194	0.58	At1g02145	2,100
APTG1b	GRMZM2G108921		None	
APTG1c	GRMZM2G103473		None	
Glycosyl Transferase Family 24 (GT24)				
EBS1b	GRMZM5G846198	0.95	None	4,272
EBS1a	GRMZM2G370162	1.97	**At1g71220**	3,308
Glycosyl Transferase Family 50 (GT50)				
PEANUT1	GRMZM2G048762	0.44	At5g22130	1,959
Glycosyl Transferase Family 57 (GT57)				
**ALG6**	GRMZM2G023884	0.62	**At5g38460**	1,659
ALG8	GRMZM2G411569		At2g44660	135
Glycosyl Transferase Family 58 (GT58)				
**ALG3**	GRMZM2G066362	0.93	**At2g47760**	2,941
Glycosyl Transferase Family 59 (GT59)				
**ALG10**	GRMZM2G056036	0.68	**At5g02410**	1,758
Glycosyl Transferase Family 64 (GT64)				
**GT64-1**	GRMZM2G121237	0.26	**At1g80290, At3g55830**	1,812
**GT64-2**	GRMZM5G889321	0.73	**At1g80290, At3g55830**	1,898
GT64-3	GRMZM2G133633		At5g04500	23
Glycosyl Transferase Family 66 (GT66)				
**STT3Aa**	GRMZM2G154165	0.23	**At5g19690**	1,592
GT66-2	GRMZM2G044096	0.47	None	6,813
**STT3Ab**	GRMZM2G060611	0.72	**At5g19690**	10,056
GT66-1	GRMZM2G040876	0.78	None	4,012
STT3B	GRMZM2G351599		At1g34130	30
**Alpha-Expansins (α-Exp)**				
**α-Exp15a**	GRMZM2G105844	0.10	At2g03090, **At1g69530, At1g26770**	2,323
**α-Exp6a**	GRMZM2G445169	0.32	**At3g55500, At2g39700, At2g28950**	716
**α-Exp6b**	GRMZM2G368886	0.50	**At3g55500, At2g39700, At2g28950**	3,613
**α-Exp13a**	GRMZM2G120724	0.67	**At3g03220**	1,764
**α-Exp13b**	GRMZM2G072121	0.88	**At3g03220**	1,485
**α-Exp8b**	GRMZM2G339122	0.92	**At5g05290, At2g40610**	3,068
α-Exp19	GRMZM5G832248	1.12	At3g29365	3,818
**α-Exp8a**	GRMZM2G361064	1.36	**At5g05290, At2g40610**	5,909
α-Exp11a	GRMZM2G074585		At1g20190	163
α-Exp12a	GRMZM2G106899		At3g15370	64
α-Exp12b	GRMZM2G139695		At3g15370	62
α-Exp20	AC219190.3_FG002		At4g38210	18
α-Exp17b	AC234190.1_FG001		At4g01630	11
α-Exp11b	GRMZM2G073373		At1g20190	10

α-Exp21	GRMZM2G126196		At5g39260	9
α-Exp17a	GRMZM2G094523		At4g01630	9
α-Exp11h	GRMZM2G414779		None	8
α-Exp15b	GRMZM2G149605		At2g03090, At1g69530, At1g26770	8
α-Exp18a	GRMZM2G127029		At1g62980, At1g12560	7
α-Exp12c	GRMZM2G030531		At3g15370	7
α-Exp11o	GRMZM2G160314		None	6
α-Exp11f	GRMZM2G469689		None	5
α-Exp11e	GRMZM2G469701		None	5
α-Exp18b	GRMZM2G066862		At1g62980, At1g12560	4
α-Exp11g	GRMZM2G173826		None	3
α-Exp11n	GRMZM2G019398		None	3
α-Exp11k	GRMZM2G374248		None	2
α-Exp11l	GRMZM2G035189		None	2
α-Exp11c	GRMZM2G146540		None	0
α-Exp11d	GRMZM2G088791		None	0
α-Exp11i	GRMZM2G332412		None	0
α-Exp11j	GRMZM2G115909		None	0
α-Exp11m	GRMZM2G064174		None	0
α-Exp11p	GRMZM2G450546		None	0
α-Exp11q	GRMZM2G303937		None	0
**α-Expansin-like Family (α-ExpL)**				
**α-ExpL2a**	GRMZM2G114322	0.52	**At4g38400, At3g45970, At3g45960**	683
**α-ExpL2b**	GRMZM2G026147	1.91	**At4g38400, At3g45970, At3g45960**	1,386
α-ExpL2c	GRMZM2G095968	8.02	At4g38400, At3g45970, At3g45960	1,951
α-ExpL2d	GRMZM2G127072		At4g38400, At3g45970, At3g45960	446
**β-Expansin-like Family (β-Exp)**				
β-Exp2k	GRMZM2G474194	0.06	None	517
**β-Exp3a**	GRMZM2G169967	0.15	**At4g28250, At2g20750**	786
β-Exp2m	GRMZM2G021427	0.80	None	669
β-Exp2i	GRMZM2G056236	0.82	None	1,620
β-Exp2h	GRMZM2G154178	0.97	None	3,692
β-Exp2n	GRMZM2G082520		None	481
β-Exp3b	GRMZM2G078279		At4g28250, At2g20750	350
β-Exp2l	GRMZM2G104013		None	322
β-Exp2c	GRMZM2G103672		None	294
β-Exp2o	GRMZM2G342246		None	272
β-Exp2j	GRMZM2G327266		None	80
β-Exp2e	GRMZM2G148485		None	52
β-Exp2d	GRMZM2G026956		None	21
β-Exp2a	GRMZM2G100931		None	12
β-Exp2f	GRMZM2G121308		None	4
β-Exp2b	GRMZM2G401983		None	3
β-Exp2g	GRMZM2G118873		None	0
**Xyloglucan endo-β-D-transglucosylase/hydrolase Family (GH16)**				
Group A				
**XTH8**	GRMZM2G174855	0.35	**At1g11545**	2,357
**XTH5**	GRMZM2G004699	0.69	**At5g13870, At2g06850**	1,054
XTH9	GRMZM2G180870		At4g03210	75
XTH11b	GRMZM2G110299		At3g48580, At3g25050, At4g13090, At4g13080	15
XTH11a	GRMZM5G845080		At3g48580, At3g25050, At4g13090, At4g13080	0
**Group B**				
**XTH15c**	GRMZM2G166944	0.38	**At3g23730, At4g14130**	26,637
XTHB8	GRMZM2G128876	0.55	None	1,207
XTHB11	GRMZM2G063566	0.73	None	1,595
XTHB10	GRMZM2G119783	1.86	None	23,810
XTHB4	GRMZM2G392125	3.45	None	9,278
XTH15d	GRMZM2G026980	101.18	At3g23730, At4g14130	3,452
XTHB3	GRMZM2G142857		None	430
XTHB9	GRMZM2G364748		None	105
XTHB2	GRMZM2G104651		None	84
XTHB1	GRMZM2G076157		None	45
XTHB5	GRMZM2G062811		None	14
XTHB6	GRMZM2G091303		None	11
XTH26a	GRMZM2G143473		At4g28850	7
XTHB7	GRMZM2G091118		None	0
XTH15a	AC210669.3_FG001		At3g23730, At4g14130	0
XTH15b	GRMZM2G413006		At3g23730, At4g14130	0
XTH26b	GRMZM2G112619		At4g28850	0
**Group C**				

**XTH28a**	GRMZM2G039919	0.02	At2g01850, **At1g14720**	1,900
**XTH31d**	GRMZM2G113761	0.15	**At2g36870**, At3g44990	2,368
XTH30c	GRMZM2G060837	0.18	At1g32170, At4g18990	900
**XTH31a**	GRMZM2G118579	0.30	**At2g36870**, At3g44990	1,303
**XTH28b**	GRMZM2G319798	0.35	At2g01850, **At1g14720**	1,919
**XTH31b**	GRMZM2G413044	0.49	**At2g36870**, At3g44990	917
**XTH30b**	GRMZM2G070271	11.05	**At1g32170, At4g18990**	1,890
XTH31c	GRMZM2G388684		At2g36870, At3g44990	359
XTH30a	GRMZM5G808290		At1g32170, At4g18990	12
**Yieldin Family**				
Yieldin2	GRMZM2G090441	0.02	None	907
CHIAh	GRMZM2G453805	43.63	At5g24090	7,649
CHIAg	AC193632.2_FG002		At5g24090	256
Yieldin1	GRMZM2G141456		None	253
CHIAa	GRMZM2G430936		At5g24090	61
CHIAe	GRMZM2G130686		None	23
CHIAc	GRMZM2G023650		At5g24090	8
CHIAf	GRMZM2G160265		None	3
CHIAb	GRMZM2G430942		At5g24090	0
CHIAd	GRMZM2G080547		None	0
**Glycosyl Hydrolase Family 9 (GH9)**				
**CEL1a**	GRMZM2G076049	0.09	**At1g70710, At1g23210**	2,249
**CEL1b**	GRMZM2G455642	0.19	**At1g70710, At1g23210**	888
GH9B8c	GRMZM2G154678	0.40	At2g32990	636
**KOR1b**	GRMZM2G003379	0.95	**At5g49720, At4g24260**	1,657
**KOR2a**	GRMZM2G178025	1.08	**At1g65610**	1,744
**KOR1a**	GRMZM2G099101	1.09	**At5g49720, At4g24260**	1,658
**KOR1c**	GRMZM2G147849	1.19	**At5g49720, At4g24260**	35,560
GH9B7a	GRMZM2G147422	1.43	At1g75680, At1g19940	21,952
KOR1d	GRMZM2G110735	1.50	At5g49720, At4g24260	67,286
GH9B8b	GRMZM2G482256	5.43	At2g32990	5,528
GH9B8a	GRMZM2G151257	7.15	At2g32990	1,494
GH9C2b	GRMZM2G143747		At4g11050, At1g64390	256
CEL5	GRMZM2G125436		At1g71380, At1g22880	135
GH9B15	GRMZM2G167669		At4g23560, At4g09740	71
GH9L2	GRMZM2G009025		None	56
CEL2	GRMZM2G331566		At4g02290, At1g02800	52
KOR2b	GRMZM2G066162		At1g65610	47
GH9B7b	GRMZM2G153987		At1g75680, At1g19940	39
GH9C1	GRMZM2G343144		At1g48930	33
GH9C2a	GRMZM2G453565		At4g11050, At1g64390	5
GH9B12	AC203408.4_FG003		At2g44570, At2g44560, At2g44550, At2g44540	
GH9L1	GRMZM2G141911		None	
**Glycosyl Hydrolase Family 17 (GH17)**				
Group A				
GH17A9	GRMZM2G019185	1.06	None	575
GH17A11	GRMZM2G137535	4.88	None	1,389
GH17A7	GRMZM2G125032	163.81	None	3,348
GH17A3	GRMZM2G123107		None	457
GH17A1	GRMZM2G380561		None	442
GH17A5	GRMZM2G062600		None	347
GH17A6	GRMZM2G061403		None	292
GH17A4	GRMZM2G065585		None	262
GH17A2	GRMZM2G088951		None	184
GH17A12	GRMZM2G014723		None	155
GH17A8	GRMZM2G433365		None	108
GH17A10	GRMZM2G041961		None	28
Group B				
**GH17B5**	GRMZM2G152638	0.30	**At4g17180**	515
GH17B1	AC233893.1_FG006	0.41	None	4,589
**GH17B6**	GRMZM2G097207	0.49	**At5g58090**, **At4g31140**	1,227
GH17B14	AC159612.1_FG007	0.56	At5g18220, At3g04010, At2g19440, At1g64760	1,017
**GH17B13**	GRMZM2G078566	34.75	At5g18220, **At3g04010**, At2g19440, At1g64760	1,208
GH17B4	GRMZM2G083222		At5g58480	353
GH17B8	GRMZM5G834199		At5g58090, At4g31140	278
GH17B7	GRMZM2G090469		At5g58090, At4g31140	269
GH17B10	GRMZM2G310739		At5g64790	116
GH17B2	AC232238.2_FG004		None	110

GH17B11	GRMZM2G083599		At5g18220, At3g04010, At2g19440, At1g64760	69
GH17B9	GRMZM2G005798		At5g64790	62
GH17B12	GRMZM2G020898		At5g18220, At3g04010, At2g19440, At1g64760	24
GH17B3	AC217887.3_FG001		None	0
Group C				
**GH17C10**	GRMZM2G172537	0.04	**At2g27500, At5g42100, At1g32860**	1,039
**GH17C11**	GRMZM2G030850	0.18	**At2g27500, At5g42100, At1g32860**	1,715
**GH17C6**	GRMZM2G046101	0.37	**At5g42720, At4g34480, At2g16230**	2,343
GH17C5	GRMZM5G858249	0.55	None	4,346
**GH17C15**	GRMZM2G148400	0.78	**At4g18340, At1g30080**	1,282
GH17C13	GRMZM2G076584	0.91	At2g27500	3,353
GH17C14	GRMZM2G177510	1.21	At2g27500	5,407
GH17C2	GRMZM2G111143	2.18	At3g46570, At3g55430, At2g39640	5,874
GH17C12	GRMZM2G127117	2.77	At2g27500	2,248
GH17C1	GRMZM2G064202		At3g46570, At3g55430, At2g39640	466
GH17C4	GRMZM2G096591		At5g42720, At4g34480, At2g16230	298
GH17C8	GRMZM2G117872		At3g15800, At2g26600	269
GH17C9	GRMZM2G008627		At3g15800, At2g26600	266
GH17C3	GRMZM2G012758		At5g42720, At4g34480, At2g16230	164
GH17C7	GRMZM2G335111		None	0
Group D				
**GH17D6**	GRMZM2G005082	0.09	**At5g56590, At4g29360**	6,864
**GH17D1**	GRMZM2G046459	0.19	**At5g55180, At4g26830**	24,412
**GH17D7**	GRMZM2G072526	0.23	**At3g13560**	2,013
**GH17D2**	GRMZM2G114140	0.23	**At5g55180, At4g26830**	2,303
**GH17D8**	GRMZM2G113420	0.32	**At3g13560**	2,250
**GH17D10**	GRMZM2G325008	0.37	**At2g01630, At1g66250**	3,864
**GH17D9**	GRMZM2G111324	0.58	**At2g01630, At1g66250**	1,259
GH17D3	GRMZM2G454550		At5g55180, At4g26830	43
GH17D5	GRMZM2G431039		At5g55180, At4g26830	36
GH17D4	GRMZM5G846916		At5g55180, At4g26830	26
Group E				
**GH17E3**	GRMZM2G458164	0.08	**At4g14080, At3g23770, At3g07320**	1,424
GH17E2	GRMZM2G179354		At4g14080, At3g23770, At3g07320	34
GH17E1	GRMZM2G000959		At4g14080, At3g23770, At3g07320	9
**Polygalacturonanases (GH28)**				
Group A				
**PGaseA3**	GRMZM2G052844	0.35	**At5g41870, At4g23820**	3,286
**PGaseA6**	GRMZM2G13982	0.37	**At3g16850**, At5g49215, **At3g06770**	2,080
**PGaseA5**	GRMZM5G882418	0.38	**At3g62110**	7,771
**PGaseA7**	GRMZM2G113815	0.43	**At3g16850**, At5g49215, **At3g06770**	4,064
**PGaseA4**	GRMZM2G057296	0.57	**At3g62110**	1,195
PGaseA12	GRMZM2G135763	1.76	At3g42950,At1g19170	4,396
PGaseA11	GRMZM2G047414	1.84	At3g42950,At1g19170	4,668
PGaseA2	GRMZM2G174598		At5g41870, At4g23820	302
PGaseA1	GRMZM2G004435		At4g33440	267
PGaseA9	GRMZM2G119494		At4g23500, At3g61490, At3g48950, At2g23900	51
PGaseA8	GRMZM2G107073		At4g23500, At3g61490, At3g48950, At2g23900	10
PGaseA10	GRMZM2G179444		At4g23500, At3g61490, At3g48950, At2g23900	3
Group C				
**PGaseC2**	GRMZM2G037431	0.06	**At1g60590, At1g10640**	1,347
**PGaseC6**	GRMZM2G467435	0.17	**At1g48100**	1,641
**PGaseC4**	GRMZM2G170388	1.06	**At1g56710, At4g01890, At1g02460**	1,103
PGaseC3	GRMZM2G002034		At1g60590, At1g10640	448
PGaseC5	GRMZM2G026804		At1g56710, At4g01890, At1g02460	242
PGX1	GRMZM2G338158		At3g26610	161
PGaseC1	GRMZM2G435380		At5g14650	94
PGaseC7	GRMZM2G098912		At1g48100	46
Group D				
**PGaseD2**	GRMZM5G831200	0.38	At1g70500, **At1g23460**	518
PGaseD3	GRMZM2G079263		At1g70500, At1g23460	344
PGaseD4	GRMZM2G079617		At1g70500, At1g23460	147
PGaseD1	GRMZM2G034835		At1g80170	132
QRT2c	GRMZM2G111609		At3g07970, At3g57510, At2g41850	119
QRT2b	GRMZM2G092746		At3g07970, At3g57510, At2g41850	112
QRT2a	GRMZM2G004500		At3g07970, At3g57510, At2g41850	6
Group E				
PGaseE3	GRMZM2G153666		At4g18180	34
PGaseE2	GRMZM2G399421		At4g18180	8
PGaseE1	GRMZM2G452150		At4g18180	6

Group F				
PGaseF15	GRMZM2G112579		None	362
PGaseF11	GRMZM2G320175		None	10
PGaseF3	GRMZM2G151755		None	9
PGaseF16	GRMZM2G027782		None	7
PGaseF13	GRMZM2G094811		None	5
PGaseF14	GRMZM2G058033		None	2
PGaseF2	GRMZM2G374375		None	0
PGaseF4	GRMZM2G386171		None	0
PGaseF5	GRMZM2G160526		None	0
PGaseF6	GRMZM2G160626		None	0
PGaseF12	GRMZM2G394259		None	0
PGaseF1	GRMZM2G030265		None	0
PGaseF7	GRMZM2G162384		None	0
PGaseF8	GRMZM2G454497		None	0
PGaseF9	GRMZM2G454608		None	0
PGaseF10	GRMZM2G418644		None	0
Group G				
PGaseG1	GRMZM2G026855	2.17	At1g65570	6,338
**Glycosyl Hydrolase Family 35 (GH35)**				
**BGAL8**	GRMZM2G127123	0.11	**At2g28470**	4,952
**BGAL1a**	GRMZM2G130375	0.18	**At3g13750**	3,937
**BGAL5a**	GRMZM2G417455	0.21	**At1g45130, At4g36360**	6,812
**BGAL14a**	GRMZM2G386824	0.23	**At4g38590, At4g35010, At2g16730**	1,187
**BGAL5b**	GRMZM2G178106	0.32	**At1g45130, At4g36360**	2,646
**BGAL1c**	GRMZM2G081583	0.38	**At3g13750**	1,719
**BGAL1d**	GRMZM2G027385	0.38	**At3g13750**	1,716
**BGAL10**	GRMZM2G038281	0.54	**At5g63810**	9,443
**BGAL9**	GRMZM2G073584	0.88	**At2g32810**	5,894
BGAL17a	GRMZM2G164676	0.97	At1g72990	2,140
BGAL17d	GRMZM2G162238		At5g20710, At2g04060, At1g31740	167
BGAL17c	GRMZM2G114756		At5g20710, At2g04060, At1g31740	115
BGAL1b	GRMZM2G121495		At3g13750	105
BGAL14c	GRMZM2G071883		At4g38590, At4g35010, At2g16730	48
BGAL14b	GRMZM2G175779		At4g38590, At4g35010, At2g16730	31
BGAL17b	GRMZM5G828603		At1g72990	0
**Pectin/Pectate Lyase Family (PL)**				
**PL5**	GRMZM2G412207	0.40	**At4g13210, At3g24670, At5g48900, At3g07010**	2,437
**PL6**	GRMZM2G131912	0.44	**At4g13210, At3g24670, At5g48900, At3g07010**	3,266
**AT59a**	GRMZM2G080056	0.52	**At5g15110, At3g01270, At2g02720, At1g14420**	584
PL4	GRMZM2G005562	2.10	At3g55140	8,496
PDE329	GRMZM2G051403	2.53	At3g55250	1,213
PL1	GRMZM2G472060		At3g53190	461
AT59e	GRMZM2G364349		At5g15110, At3g01270, At2g02720, At1g14420	24
AT59d	GRMZM2G323418		At5g15110, At3g01270, At2g02720, At1g14420	19
AT59c	AC194852.3_FG007		At5g15110, At3g01270, At2g02720, At1g14420	17
PL3	GRMZM2G126077		At3g09540	7
PL2	GRMZM2G139396		At5g09280	3
AT59b	GRMZM2G129635		At5g15110, At3g01270, At2g02720, At1g14420	0
**Rhamnogalacturonan-I lyase Family (RGIL)**				
**RGlyase1**	GRMZM2G154124	0.36	**At1g09890**	2,215
RGlyase2	GRMZM2G015792		At4g38030, At2g22620, At4g37950	133
RGlyase3	GRMZM2G175698		At4g38030, At2g22620, At4g37950	34
RGlyase4	GRMZM2G450771		At4g38030, At2g22620, At4g37950	10
**Pectin Methylesterase (PME)**				
Group A				
**PME5**	GRMZM2G162333	0.04	**At5g47500**	2,261
**PMEL8**	GRMZM2G167637	0.17	**At5g64640, At5g09760**	1,475
**PME31b**	GRMZM2G178916	1.60	**At3g29090**	1,380
PMEL9	GRMZM2G004927		At3g17060	487
PME31a	GRMZM2G019411		At3g29090	417
PMEL13	GRMZM2G043943		At5g19730	402
PMEL15	GRMZM2G177940		At5g19730	364
QRT1	GRMZM2G037411		At5g55590	181
PMEL17	GRMZM2G455564		At1g05310	107
PMEL14	GRMZM2G120779		**At5g19730**	61
PMEL11	GRMZM2G431856		At5g07430, At5g07420	48

PMEL10	GRMZM2G121278		At2g47280, At2g19150	48
PMEL12	GRMZM2G382557		At5g07430, At5g07420	3
PMEL16	GRMZM2G318299		At2g36710, At2g36700	0
Group B				
VGDH2b	GRMZM2G352359		At3g62170, At2g47040, At2g47030	36
VGDH2d	GRMZM2G012328		At3g62170, At2g47040, At2g47030	31
VGDH2c	GRMZM2G125356		At3g62170, At2g47040, At2g47030	18
PMEL7	GRMZM2G314663		At4g33230, At2g26450	17
PMEL5	GRMZM2G137676		At4g33230, At2g26450	10
PMEL4	GRMZM2G060400		At4g33230, At2g26450	8
PMEL3	GRMZM2G043415		At4g33230, At2g26450	7
PME58b	GRMZM2G112984		At3g05610, At5g27870	5
PMEL6	GRMZM2G062996		At4g33230, At2g26450	4
VGDH2a	GRMZM2G128549		At3g62170, At2g47040, At2g47030	3
PME58a	GRMZM2G321870		At3g05610, At5g27870	0
Group D				
**PME12**	GRMZM2G046618	0.10	**At2g26440**	516
**PMEL1**	GRMZM2G008593	0.32	At5g53370, **At3g49220**	1,109
**PME2b**	GRMZM2G025182	0.72	**At3g14310, At1g53830**	2,473
**PME2a**	GRMZM2G136106	0.88	**At3g14310, At1g53830**	2,409
PME44	GRMZM2G175499	2.21	At4g33220, At3g43270	1,454
PMEL2	GRMZM2G017555		At3g05620	412
PME35	GRMZM2G158240		At3g59010, At2g43050	183
PME41b	GRMZM2G119864		At2g47550, At4g02330, At1g02810	35
PME41a	GRMZM2G128682		At2g47550, At4g02330, At1g02810	3
**Pectin Acetylesterase Family**				
**PAE7**	GRMZM2G156365	0.20	**At5g45280, At4g19410**	5,680
PAEL5	GRMZM2G336879	0.38	None	608
**PAEL1**	GRMZM2G141873	0.47	**At3g09410**	1,397
PAEL8	GRMZM2G160569	0.51	None	5,751
PAEL3	GRMZM2G329002	6.66	None	1,580
PAEL2	GRMZM2G016561		At3g09410	386
PAE8	AC204604.3_FG008		At4g19420	284
PAEL7	GRMZM2G164134		None	277
PAE9	GRMZM2G117999		At5g23870	272
PAEL6	GRMZM2G066500		None	131
PAEL4	GRMZM2G418635		None	1
**Arabinogalactan-Protein/Fasciclin Family**				
**FLA10a**	GRMZM2G003752	0.15	**At3g60900, At2g45470**	1,338
AGPa	AC209784.3_FG007	0.16	None	11,880
FLA4b	GRMZM2G035933	0.20	At3g46550	647
FLA2a	GRMZM2G003165	3.87	At5g55730, At4g12730	1,472
**FLA11**	GRMZM2G177242	6.62	**At5g03170**, At5g60490	91,930
AGP24	AC213621.5_FG002		At5g40730	335
FLA2c	GRMZM2G148534		At5g55730, At4g12730	193
FLA2b	GRMZM2G144610		At5g55730, At4g12730	30
FLA10b	GRMZM2G065718		At3g60900, At2g45470	2
FLA4a	GRMZM2G421415		At3g46550	0
**Prolyl-4-hydroxylases**				
**P4Hh**	GRMZM2G168506	0.28	**At3g28480**	2,158
**P4Hd**	GRMZM2G348578	0.40	**At4g33910**	934
**P4H-2**	GRMZM2G459063	0.48	**At5g18900, At3g06300**	2,307
**P4Hg**	GRMZM2G520535	0.67	**At3g28480**	2,032
**P4Ha**	GRMZM2G145061	0.74	**At1g20270**	1,640
**P4Hb**	GRMZM2G025867	0.84	**At1g20270**	4,357
**P4H-1**	GRMZM2G028004	0.89	**At2g43080**	1,554
**P4Hc**	GRMZM5G855891	1.07	**At1g20270**	1,235
P4He	GRMZM5G843555		None	357
P4Hf	GRMZM2G054224		None	87
**Receptor-like Kinase Family**				
CRK4c	GRMZM2G168588	0.02	None	2,237
CRK4i	GRMZM2G058394	0.05	None	789
CRK4j	GRMZM2G476523	0.13	None	5,284
CRK4s	GRMZM2G148536	0.18	None	1,790
CRK4r	GRMZM2G151589	0.18	None	1,608
CRK4f	GRMZM2G154301	0.23	None	808
CRK4e	GRMZM2G048194	0.36	None	3,317

CRK4h	GRMZM2G064605	0.40	None	2,692
CRK4v	GRMZM2G049057	0.51	None	682
CRK4d	GRMZM2G421463	0.54	None	762
CRK4u	GRMZM2G138896	0.63	None	632
CRK4z	GRMZM2G477139	0.83	None	3,710
CRK4q	GRMZM2G149798	1.08	None	747
CRK4p	GRMZM2G346861	1.57	None	902
CRK4x	GRMZM2G159110	2.36	None	5,292
CRK4b	GRMZM2G410991	3.26	None	3,041
CRK4ac	GRMZM2G036826	21.38	None	4,764
CRK4a	AC205471.4_FG003		None	357
CRK4y	GRMZM2G149809		None	302
CRK4n	GRMZM2G038846		None	295
CRK4k	GRMZM2G125918		None	238
CRK4t	GRMZM2G154449		None	163
CLVc	GRMZM2G166413		At1g65380	94
CRK4l	GRMZM2G340534		None	73
CRK4o	GRMZM2G393507		None	18
CRK4w	GRMZM2G023655		None	16
CLVb	AC204763.2_FG001		At1g65380	15
CLVa	GRMZM2G011401		At1g65380	10
CRK4aa	GRMZM2G038490		None	8
CRK4ab	GRMZM2G086410		None	7
CRK4g	GRMZM2G004617		None	0
CRK4m	GRMZM2G100747		None	0
**Proteases**				
Aspartyl Group				
Aspartyl5	GRMZM2G335978	0.67	At5g10080	1,722
**Aspartyl4**	GRMZM2G177575	0.68	**At4g35880**	2,389
Aspartyl3	GRMZM2G420910	1.24	At4g35880	695
Aspartyl7	GRMZM2G133029	3.01	At1g08210	9,115
Aspartyl6	GRMZM2G411333	5.23	At1g05840	1,331
Aspartyl2	GRMZM2G168115	14.87	At4g35880	3,153
Aspartyl1	AC190609.3_FG001		None	32
Cysteine Group				
**MPL1a**	GRMZM2G047533	0.29	**At5g14180**	1,128
**Cys1**	GRMZM2G098298	1.58	**At3g43960**	25,126
**MPL1e**	GRMZM2G027825	2.99	**At3g52370**	18,210
**MPL1d**	GRMZM2G329181	3.54	**At3g52370**	19,058
MPL1b	GRMZM2G057136		At5g14180	150
MPL1c	GRMZM2G322892		At5g14180	122
Metallo Group				
**MPb**	GRMZM2G110220	1.13	**At1g59970, At1g70170**, At1g24140	1,716
MPa	GRMZM2G317386		At4g16640, At2g45040	0
Other Proteins				
OP32	GRMZM2G340065	0.13	None	758
OP33	GRMZM2G010435	0.21	None	1,512
OP27	GRMZM2G066326	0.22	None	7,782
OP26	GRMZM2G367701	0.65	None	3,263
OP36	AC155377.1_FG001	1.19	None	8,935
OP30	GRMZM2G073465	1.53	None	5,186
OP28	GRMZM2G006377	1.57	None	4,629
OP29	GRMZM2G166281	1.90	None	661
OP25	GRMZM2G180926		None	358
OP22	GRMZM2G456217		None	232
OP31	GRMZM2G070571		None	223
OP5	GRMZM2G045706		None	184
OP34	GRMZM2G150256		None	180
OP1	GRMZM2G014526		None	85
OP2	GRMZM2G097286		None	53
OP21	GRMZM2G099765		None	28
OP6	GRMZM2G130053		None	24
OP3	GRMZM2G060090		None	20
OP19	GRMZM2G077479		None	19
OP16	GRMZM2G070011		None	16
OP13	GRMZM2G137690		None	15
OP7	GRMZM2G363926		None	14
OP14	GRMZM2G334321		None	13
OP4	GRMZM2G160447		None	11
OP8	GRMZM2G397965		None	8

OP15	GRMZM2G019742		None	8
OP24	GRMZM2G035045		None	8
OP35	GRMZM2G150276		None	4
OP9	GRMZM2G028862		None	2
OP12	AC225716.2_FG003		None	2
OP20	GRMZM2G020657		None	1
OP10	GRMZM2G095628		None	0
OP11	GRMZM2G165086		None	0
OP17	GRMZM2G098102		None	0
OP18	GRMZM2G166870		None	0
OP23	AC209810.3_FG002		None	0
**GPI-anchored proteins**				
**Cobra-like proteins**				
COBL4a	GRMZM2G167497	0.40	At5g15630	1,485
**COBL9b**	GRMZM2G377215	0.56	**At5g49270, At4g16120, At3g16860**	7,886
COBRAb	GRMZM5G826714	1.14	None	61,004
**COBL4b**	GRMZM2G109326	4.47	**At5g15630**	11,909
COBL1	GRMZM2G167520		At3g29810, At3g02210	131
COBL6	GRMZM2G465188		At1g09790	25
COBL10	GRMZM2G056627		At4g27110, At3g20580	10
COBRAa	GRMZM2G071970		None	1
COBL9a	GRMZM2G353276		At5g49270, At4g16120, At3g16860	0
**SKU-like proteins**				
SKU2	GRMZM2G402584	0.03	None	1,326
SKU3	GRMZM2G049693	0.06	None	2,765
SKU10	GRMZM2G076225	0.09	None	931
SKU4	GRMZM2G172642	0.23	None	1,237
SKU7	GRMZM2G043301	0.29	None	1,108
SKU9	GRMZM2G076985	0.73	None	4,959
SKU1	GRMZM2G438386	0.91	None	1,792
SKU5	GRMZM2G077317		None	345
SKU6	AC177897.2_FG002		None	251
SKU12	GRMZM2G142584		None	123
SKU13	GRMZM2G129064		None	14
SKU8	GRMZM2G360529		None	10
SKU11	GRMZM2G157929		None	6
**Monolignol synthesis**				
Phenylalanine Ammonia Lyase (PAL)				
PAL6	GRMZM2G118345	0.57	None	743
PAL4	GRMZM2G081582	2.53	None	5,605
PAL3	GRMZM2G441347	3.95	None	1,840
PAL5	GRMZM2G029048	4.10	None	33,864
PAL1	GRMZM2G160541	5.06	None	27,762
PAL8	GRMZM2G334660	5.28	None	2,172
PAL10	GRMZM2G063917	5.57	None	1,138
PAL2	GRMZM2G074604	6.34	None	137,599
PAL9	GRMZM2G170692	7.62	None	8,478
PAL7	GRMZM2G153871		None	71
4-Coumarate CoA Ligase (4-CL)				
**4CLL8a**	GRMZM5G805585	0.20	**At5g63380**	968
**4CLL4**	GRMZM2G096020	1.41	**At1g20480**	3,246
**4CLL7**	GRMZM2G122787	1.87	**At4g05160**	1,828
4CLL8c	GRMZM2G091643	3.12	At5g63380	959
**4CL4**	GRMZM2G055320	3.58	**At1g51680, At3g21240, At3g21230**	10,101
4CLL6	GRMZM2G433624	13.55	At4g19010	2,023
4CL3	GRMZM2G054013		At1g65060	482
4CLL8b	GRMZM2G019746		None	248
4CLL5a	GRMZM2G014651		At1g62940	8
4CLL5b	GRMZM2G145179		At1g62940	8
Cinnamyl-CoA Reductase (CCR)				
**CCRL1b**	GRMZM2G034069	0.17	**At1g76470, At2g33600**, At2g33590	4,375
**CCRL5a**	GRMZM2G110881	0.21	At5g58490	519
**CCRL1c**	GRMZM2G033555	0.33	**At1g76470, At2g33600**, At2g33590	10,953
**CCR1b**	GRMZM2G131836	0.42	**At1g15950, At1g80820**	853
CCR1f	GRMZM2G179981	0.86	None	12,097
CCRL1e	GRMZM2G050076	2.35	None	1,861
CCR1a	GRMZM2G131205	2.66	At1g15950, At1g80820	32,123
CCRL5b	GRMZM2G107076	2.80	At5g58490	2,793
CCRL1a	GRMZM2G009681	3.88	At1g76470, At2g33600, At2g33590	2,524

CCR1g	GRMZM2G168893		None	292
CCR1h	GRMZM2G004683		None	175
CCR1e	GRMZM2G146031		None	138
CCR1c	GRMZM2G099420		None	82
CCRL1d	GRMZM2G016836		None	57
CCRL2	GRMZM2G141350		At2g02400	27
CCRL1f	AC234526.1_FG005		None	15
CCR1d	GRMZM2G017285		None	13
CCRL1g	GRMZM2G078480		None	10
Hydroxycinnamoyl-CoA Transferase (HCT)				
**HCTL9**	GRMZM2G124066	0.10	**At1g27620**	1,045
SHTb	GRMZM2G051005	0.18	None	613
**ASFTa**	GRMZM2G034360	0.29	**At5g41040**	4,486
**HCTL4b**	GRMZM2G115422	0.31	**At5g01210**	1,138
**DCR**	GRMZM2G179703	0.36	**At5g23940**	2,312
SHTf	GRMZM2G156816	0.59	None	2,247
SHTi	GRMZM2G178769	1.29	None	752
CHATh	GRMZM2G050450	1.36	None	699
**CHATc**	GRMZM2G129266	1.54	**At5g17540, At3g03480**	1,548
HCTL4a	GRMZM2G094017	2.38	At5g01210	1,608
SHTd	GRMZM2G156296	2.60	None	1,712
**HCTa**	GRMZM2G158083	5.56	**At5g48930**	9,089
**HCTb**	GRMZM2G035584	6.66	**At5g48930**	36,861
HCTL3b	GRMZM2G156004	10.99	At1g78990, At1g32910	2,534
CHATk	GRMZM2G107851		None	390
HCTL4d	GRMZM2G124815		At5g01210	288
HCTL7b	GRMZM2G035023		At5g38130	245
SHTh	GRMZM2G132678		None	224
SHTj	GRMZM2G107211		None	204
HCTL7c	GRMZM2G015793		At5g38130	201
SHTc	GRMZM2G131165		None	112
SHTe	GRMZM2G177349		None	87
CHATd	GRMZM2G005046		At5g17540, At3g03480	79
SHTg	GRMZM2G176446		None	59
HCTL3a	GRMZM2G070468		At1g78990, At1g32910	22
CHATa	GRMZM2G037591		At5g17540, At3g03480	16
CHATf	GRMZM2G043154		None	16
CHATl	GRMZM2G154216		None	9
HCTL7a	GRMZM2G064969		At5g38130	5
SHTa	GRMZM2G127251		None	5
ASFTc	GRMZM2G417382		At5g41040	5
HCTL4c	GRMZM2G165192		At5g01210	4
CHATg	GRMZM2G147908		None	4
CHATe	GRMZM2G151553		None	3
CHATb	GRMZM2G122503		At5g17540, At3g03480	2
ASFTb	GRMZM2G168499		At5g41040	0
CHATi	GRMZM2G063139		None	0
CHATj	GRMZM2G446652		None	0
p-Coumarate 3-Hydroxylase (C3H)				
C3H1a	GRMZM2G138074	1.26	At2g40890	2,777
**C3H1b**	GRMZM2G140817	4.40	**At2g40890**	20,305
Cinnamate 4-Hydroxylase (C4H)				
**C4Hb**	GRMZM2G147245	2.20	**At2g30490**	4,222
**C4Hc**	GRMZM2G139874	3.06	**At2g30490**	2,479
C4Ha	GRMZM2G010468		At2g30490	140
C4Hd	GRMZM2G028677		At2g30490	40
Ferulate 5-Hydroxylase (F5H)				
**F5Ha**	GRMZM2G100158	9.59	**At4g36220**, At5g04330	1,594
F5Hb	GRMZM5G830329		At4g36220, At5g04330	189
Caffeoyl-CoA O-Methyltransferase				
CCoAOMT1a	GRMZM2G127948	0.20	At4g34050	865
CCoAOMT1d	GRMZM2G033952	0.38	None	1,139
**OMTF3**	GRMZM2G077486	0.68	At3g61990, **At3g62000**	659
CCoAOMT1c	GRMZM2G004138	2.05	None	844
CCoAOMT1e	GRMZM2G332522	3.44	None	25,035
**CCoAOMT1b**	GRMZM2G099363	5.16	**At4g34050**	55,502
Caffeic acid O-methyltransferase				
ASMT	GRMZM2G082007		At4g35150, At4g35160	232
COMT1b	GRMZM2G423331		At5g54160	30
COMT1a	AC196475.3_FG004		At5g54160	0
Cinnamoyl alcohol dehydrogenases (CAD)				

CADa	GRMZM2G700188	0.78	None	8,104
**CAD9a**	GRMZM2G090980	1.64	**At1g72680**	3,672
**CAD6**	GRMZM5G844562	3.78	**At3g19450, At4g34230**	34,747
CADb	GRMZM2G118610	4.95	None	524
CAD9c	GRMZM2G167613	18.90	At1g72680	3,077
CADc	GRMZM2G046070		None	325
CAD9b	GRMZM2G443445		At1g72680	272
**Peroxidase Family**				
Group A				
**PRDA3**	GRMZM2G419953	0.13	**At4g11290, At3g21770, At1g05260**	883
**PRDA2**	GRMZM2G136158	0.20	**At2g39040**	1,661
**PRDA1**	GRMZM2G144648	0.82	**At2g39040**	852
PRDA4	GRMZM2G085967	4.12	At4g11290, At3g21770, At1g05260	32,834
PRDA12	GRMZM2G089895	10.83	None	2,698
PRDA11	GRMZM2G410175		None	173
RCI3	GRMZM2G320269		At5g15180, At3g01190, At1g05240	89
PRDA5	GRMZM2G448051		None	34
PRDA13	GRMZM2G138450		None	22
PRDA6	GRMZM2G070603		None	7
PRDA8	GRMZM2G104109		None	5
PRDA7	GRMZM2G370928		None	2
PRDA9	GRMZM2G150780		None	0
PRDA10	GRMZM2G055487		None	0
PRDA14	GRMZM2G404676		None	0
Group B				
PRDB8	GRMZM2G127945		None	65
PRDB1	GRMZM2G101221		At4g25980, At1g77100	59
PRDB4	GRMZM2G122816		At5g17820, At3g03670, At4g26010, At1g34510	39
PRDB7	GRMZM2G103169		None	13
RHS18	GRMZM2G068699		None	8
PRDB3	GRMZM2G061230		At4g25980, At1g77100	6
PRDB5	GRMZM2G063435		At5g17820, At3g03670, At4g26010, At1g34510	1
PRDB6	GRMZM2G116452		None	1
PRDB2	GRMZM2G081928		At4g25980, At1g77100	0
Group C				
**PRX72**	GRMZM2G023840	0.27	**At5g66390**	2,591
**PRDC3**	GRMZM2G089982	0.32	**At5g66390**	13,735
PRDC4	GRMZM2G041308	0.57	At5g66390	5,046
PRDC5	GRMZM5G843748	2.96	At2g22420	654
PRDC1	GRMZM2G069093		None	27
PRDC2	GRMZM2G407740		None	0
Group D				
PRDD5	GRMZM2G080689	0.05	None	1,119
PRDD6	GRMZM2G405581	0.18	None	866
PRDD32	GRMZM2G108153	0.41	None	692
**PRDD28**	GRMZM2G095404	0.64	**At1g68850**	1,046
PRDD7	GRMZM2G405459	0.94	None	1,591
**PRDD26**	GRMZM2G150893	1.00	**At5g19880, At5g06730, At5g06720**	3,473
PRX52b	GRMZM2G171078	1.02	At5g05340	1,339
PRDD24	GRMZM2G116846	1.71	At5g19880, At5g06730, At5g06720	5,143
PRDD10	GRMZM2G020523	1.85	None	4,882
PRDD15	GRMZM2G126261	8.84	None	11,769
PRDD12	GRMZM2G427815	54.86	None	1,415
PRDD17	GRMZM2G042347		None	420
PRDD8	GRMZM2G382379		None	372
PER12	GRMZM2G103342		At1g71695	334
PRDD1	GRMZM2G471357		None	296
PRDD25	GRMZM2G088765		None	263
PRDD23	GRMZM2G117365		None	241
PRX52a	AC197758.3_FG004		At5g05340	241
PRDD13	GRMZM2G133475		None	198
PRDD34	GRMZM2G313184		At4g33420	184
PER64	GRMZM2G160327		At5g42180	87
PRDD2	GRMZM2G117706		None	67
PRDD11	GRMZM2G025441		None	47
PRDD14	GRMZM2G450717		None	39
PRDD20	GRMZM2G116902		At5g19890	37
PRDD31	GRMZM2G108219		None	19
PRDD33	GRMZM2G136525		None	16
PRDD22	GRMZM2G116823		At5g19880, At5g06730, At5g06720	14

PRDD16	GRMZM2G015280		None	12
PRDD27	GRMZM2G033985		At4g16270	12
PRDD29	GRMZM2G108123		None	12
PRDD21	GRMZM2G130904		At5g19890	7
PRDD3	GRMZM2G035506		None	6
PRDD4	GRMZM2G380247		None	5
PRDD9	GRMZM2G443885		None	5
PRDD30	GRMZM2G108207		None	5
PRDD19	GRMZM2G129543		At5g19880, At5g06730, At5g06720	3
PRDD18	GRMZM2G097934		None	0
Group E				
**APX5b**	GRMZM2G004211	0.06	**At4g35970**	1,962
**PRDE5**	GRMZM2G047456	0.25	**At5g14130**	1,704
**APX5f**	GRMZM2G014397	0.53	**At4g35970**	3,019
**APX5c**	GRMZM2G140667	0.56	**At4g35970**	16,585
**APX5e**	GRMZM2G054300	0.76	**At4g35970**	29,408
PRDE15	GRMZM2G080183	0.79	None	1,023
**APX5a**	GRMZM2G460406	1.32	**At4g35970**	12,502
APX5g	GRMZM2G006791	1.82	At4g35970	4,343
APX5d	GRMZM2G137839	1.94	At4g35970	68,200
PRDE8	GRMZM2G136534	4.88	At5g14130	9,499
PRDE13	GRMZM2G133434	35.68	None	692
PRDE9	GRMZM2G047656		At5g14130	201
PRDE14	AC210003.2_FG004		None	181
PRDE2	GRMZM2G442008		At1g24110	117
PRDE1	GRMZM2G408963		At1g24110	60
PRDE10	GRMZM2G176085		At5g14130	47
PRDE4	AC230013.2_FG002		At2g34060	40
PRDE7	GRMZM2G048775		None	9
PRDE12	GRMZM2G061776		None	7
PRDE3	GRMZM2G439422		At1g24110	3
PRDE6	GRMZM2G136042		None	0
PRDE11	GRMZM2G142011		None	0
Group F				
PRDF3	GRMZM2G010640		At1g30870	18
PRDF1	GRMZM2G150134		At3g42570, At3g17070	3
PRDF2	GRMZM2G067096		At4g33870	3
Group G				
PRDG4	GRMZM2G104394	0.15	None	1,366
PRDG1	GRMZM2G341934	0.38	None	4,198
PRDG3	GRMZM2G061088	0.59	None	596
PRDG9	GRMZM2G394500	8.16	None	5,047
PRDG7	GRMZM2G135108	9.48	None	929
PRDG6	GRMZM2G450233	58.17	None	4,937
PRDG5	GRMZM2G107228		None	201
PRDG16	AC205413.4_FG001		None	171
PRDG12	GRMZM2G365774		None	86
PRDG14	GRMZM2G076562		None	47
PRDG29	GRMZM2G150731		None	19
PRDG25	GRMZM2G044049		None	17
PRDG8	GRMZM2G012263		None	17
PRDG22	GRMZM2G427954		None	14
PRDG18	GRMZM2G022740		None	10
PRDG15	GRMZM2G177792		None	8
PRDG13	GRMZM2G029479		None	7
PRDG20	GRMZM2G129935		None	7
PRDG24	GRMZM2G427937		None	5
PRDG28	GRMZM2G004984		None	5
PRDG11	GRMZM2G060721		None	4
PRDG30	GRMZM2G029144		None	3
PRDG10	GRMZM2G160062		None	1
PRDG26	GRMZM2G040638		None	1
PRDG2	GRMZM2G149273		None	0
PRDG19	GRMZM2G451097		None	0
PRDG31	GRMZM2G156257		None	0
PRDG17	GRMZM2G050829		None	0
PRDG21	GRMZM2G027217		None	0
PRDG23	GRMZM2G437207		None	0
PRDG27	GRMZM2G168073		None	0

Laccases

Lac7h	GRMZM2G141376	0.12	None	3,427
**Lac7b**	GRMZM2G309594	0.57	**At3g09220**, At5g01050, **At5g01040**	2,060
Lac5c	GRMZM2G132169	0.68	At5g05390, At2g40370	2,060
Lac17a	GRMZM2G072808	0.74	At5g60020	720
Lac2b	GRMZM2G146152	1.07	At2g29130	3,197
Lac17b	GRMZM2G447271	1.51	At5g60020	1,477
**Lac17c**	GRMZM2G164467	4.54	**At5g60020**	7,999
**Lac17d**	GRMZM2G072780	4.55	**At5g60020**	8,010
**Lac2c**	GRMZM2G367668	7.68	**At2g29130**	9,947
Lac7d	GRMZM5G842071	10.06	At3g09220, At5g01050, At5g01040	782
**Lac2a**	GRMZM2G305526	14.58	**At2g29130**	21,219
Lac7j	GRMZM2G163535		None	401
Lac5b	GRMZM2G336337		At5g05390, At2g40370	145
Lac7k	GRMZM2G064106		None	118
Lac5a	GRMZM2G388587		At5g05390, At2g40370	103
Lac7g	GRMZM5G870184		None	94
Lac3	GRMZM5G814718		At5g07130, At2g30210	55
Lac7f	GRMZM2G166857		None	27
Lac7c	GRMZM2G094375		At3g09220, At5g01050, At5g01040	21
Lac15a	GRMZM2G169033		At5g48100	20
Lac7a	GRMZM2G400390		At3g09220, At5g01050, At5g01040	19
Lac7i	GRMZM2G386170		None	8
Lac15b	GRMZM2G320786		At5g48100	4
Lac7e	GRMZM2G140527		None	2
